# The circular RNA *Edis* regulates neurodevelopment and innate immunity

**DOI:** 10.1371/journal.pgen.1010429

**Published:** 2022-10-27

**Authors:** Xiao-Peng Xiong, Weihong Liang, Wei Liu, Shiyu Xu, Jian-Liang Li, Antonio Tito, Julia Situ, Daniel Martinez, Chunlai Wu, Ranjan J. Perera, Sheng Zhang, Rui Zhou

**Affiliations:** 1 Tumor Initiation and Maintenance Program; NCI-Designated Cancer Center, Sanford Burnham Prebys Medical Discovery Institute, La Jolla, California, United States of America; 2 Development, Aging and Regeneration Program, Sanford Burnham Prebys Medical Discovery Institute, La Jolla, California, United States of America; 3 Department of Medicine, Johns Hopkins University School of Medicine, Baltimore, Maryland, United States of America; 4 Department of Biological Chemistry, Johns Hopkins University School of Medicine, Baltimore, Maryland, United States of America; 5 Department of Oncology, Johns Hopkins University School of Medicine, Baltimore, Maryland, United States of America; 6 Cancer and Blood Disorders Institute. Johns Hopkins All Children’s Hospital, Saint Petersburg, Florida, United States of America; 7 Institute for Fundamental Biomedical Research, Johns Hopkins All Children’s Hospital, Saint Petersburg, Florida, United States of America; 8 The Brown Foundation Institute of Molecular Medicine, Department of Neurobiology and Anatomy, McGovern Medical School at the University of Texas Health Science Center at Houston, Houston, Texas, United States of America; 9 National Institute of Environmental Health Sciences, Durham, North Carolina, United States of America; 10 Neuroscience Center of Excellence, Department of Cell Biology and Anatomy, Louisiana State University Health Sciences Center, New Orleans, Louisiana, United States of America; 11 Programs in Genetics & Epigenetics and Neuroscience, the University of Texas MD Anderson Cancer Center UTHealth Graduate School of Biomedical Sciences, Houston, Texas, United States of America; University of Massachusetts, Worcester, UNITED STATES

## Abstract

Circular RNAs (**circRNAs**) are widely expressed in eukaryotes. However, only a subset has been functionally characterized. We identify and validate a collection of circRNAs in *Drosophila*, and show that depletion of the brain-enriched circRNA *Edis* (*circ_Ect4*) causes hyperactivation of antibacterial innate immunity both in cultured cells and *in vivo*. Notably, *Edis* depleted flies display heightened resistance to bacterial infection and enhanced pathogen clearance. Conversely, ectopic *Edis* expression blocks innate immunity signaling. In addition, inactivation of *Edis in vivo* leads to impaired locomotor activity and shortened lifespan. Remarkably, these phenotypes can be recapitulated with neuron-specific depletion of *Edis*, accompanied by defective neurodevelopment. Furthermore, inactivation of *Relish* suppresses the innate immunity hyperactivation phenotype in the fly brain. Moreover, we provide evidence that *Edis* encodes a functional protein that associates with and compromises the processing and activation of the immune transcription factor Relish. Importantly, restoring *Edis* expression or ectopic expression of *Edis*-encoded protein suppresses both innate immunity and neurodevelopment phenotypes elicited by *Edis* depletion. Thus, our study establishes *Edis* as a key regulator of neurodevelopment and innate immunity.

## Author summary

Circular RNAs (circRNAs) are a new group of noncoding and regulatory RNAs that are widely expressed. However, their physiological functions are underexplored. We identify and validate a collection of circRNAs in the fruitfly *Drosophila melanogaster*, and show that depletion of the brain-enriched circRNA *Edis* causes hyperactivation of antibacterial innate immunity both in cultured cells and in the organism. Notably, *Edis* depleted flies display heightened resistance to bacterial infection and enhanced pathogen clearance. Conversely, *Edis* overexpression blocks innate immunity activation. In addition, reducing *Edis* levels throughout the fly body causes impaired mobility and shortened lifespan. Remarkably, these phenotypes can be recapitulated with neuron-specific reduction in *Edis* levels, accompanied by defective neurodevelopment. Furthermore, inactivation of *Relish*, which encodes an immune transcription factor, suppresses the innate immunity hyperactivation phenotype in the fly brain. Moreover, we provide evidence that *Edis* encodes a functional protein that associates with and compromises the processing and activation of Relish. Importantly, restoring *Edis* RNA levels or expression of *Edis*-encoded protein suppresses both innate immunity and neurodevelopmental phenotypes elicited by *Edis* depletion. Thus, our study establishes *Edis* as a key regulator of neurodevelopment and innate immunity.

## Introduction

The innate immune response, the first line of defense against invading microbes, is triggered not only by pathogen-derived molecules but also by endogenous signals generated by stressed and injured cells [1]. While effective control of pathogens or response to endogenous stress signals depend on rapid and robust activation of innate immunity signaling pathways, prolonged or aberrant activation can lead to pathological conditions such as autoimmunity and cancer [2,3]. Recently, a growing body of evidence has started to link chronic and excessive inflammatory responses to aging and neurodegeneration. For example, aging brains display a sustained increase in innate immunity activity while long-term use of anti-inflammatory drugs substantially reduces the risk for Alzheimer’s disease [4] and Parkinson’s Disease [5]. Thus, proper regulation of immune responses in the brain is especially important for aging and aging-related brain diseases. These findings underscore the importance of a thorough understanding of the regulatory mechanism that controls the magnitude and duration of innate immunity signaling in physiological and pathological settings.

The fruit fly *Drosophila melanogaster* has been pivotal in elucidating the conserved innate immunity pathways [6]. For example, in response to infection by RNA viruses, the RNA interference machineries are mobilized to control viral genome replication [7–12]. In addition, upon systemic Gram-negative bacterial infection, the *im*mune *d*eficiency (**IMD**) signaling pathway is activated [13–17], which involves the trans-membrane *p*eptido*g*lycan-*r*ecognition *p*rotein (**PGRP-LC**), adaptor proteins IMD and dFADD, the caspase Dredd, and the MAP3K complex dTAK1/dTAB2 [18–23]. This leads to the activation of the *Iκ*B *k*inase Ird5/Kenny complex (**IKK**) and subsequent phosphorylation and endoproteolytic cleavage of the composite NF-κB protein Relish [24–28]. The N-terminal fragment of Relish translocates to the nucleus and induces the transcription of genes encoding potent antibacterial peptides, such as *diptericin* [23,29]. In response to Gram-positive bacterial or fungal infection, the Toll pathway is activated, resulting in nuclear translocation of another NF-κB protein Dif and activation of genes encoding anti-fungal peptides, such as *Drosomycin* [30–33]. These *a*nti*m*icrobial *p*eptides (**AMP**s) are produced primarily in the fat body and secreted into the hemolymph to mount a systemic humoral defense against invading pathogens. In addition, AMPs can be made locally by cells in the digestive tract or trachea that are exposed to microbes.

Reminiscent of the observations made in human brain diseases, aberrant activation of innate immunity in *Drosophila* can similarly lead to neurodegeneration. For example, knockdown of *ATM* (ataxia-telangiectasia mutated) in glial cells causes elevated activation of innate immunity and death of neuronal and glial cells [34]. In addition, mutations in *Dnr1*, a negative regulator of innate immunity signaling, leads to heightened expression of innate immunity response genes and accelerated neurodegeneration [35]. These findings highlight the importance of proper control of immune response in neural development and healthy aging, and validate the relevance of fly model for studying the still-unclear link between immune pathways and neurodegenerative diseases.

Much work over the past two decades has solidified the notion that regulatory and non-coding RNAs play a key role in regulating innate immunity. For example, virally derived small interfering RNAs can serve as guides for the RNA interference machinery to target viral RNAs for destruction [7–11,33,36–38]. Also, by targeting mRNAs encoding signaling proteins or inflammatory cytokines, *mi*cro*RNA*s (**miRNA**s) can profoundly impact the magnitude and duration of the inflammatory response [39–43]. Recently *circ*ular *RNA*s (**circRNA**s), which are products of “head-to-tail” back-splicing events, have been discovered in eukaryotes and constitute the latest addition to the regulatory RNA collection [44–55]. Select circRNAs have been functionally characterized. For example, the mouse circRNA *CDR1as/CiRS-7* regulates *miR-7* expression and impacts brain development [46,49,56]. In addition, the circRNA *SRY* plays a prominent role in male sex determination [46,49,56]. Furthermore, select intron-containing circRNAs can interact with U1 snRNP and promote host gene transcription in the nucleus [57]. Moreover, circRNAs can regulate gene expression by competing with linear splicing [58]. Lastly, some circRNAs can give rise to functional polypeptides, thereby expanding the complexity of the proteome [59,60]. Recently a group of hairpin-containing circRNAs have been shown to modulate innate immunity in mammalian cells [61]. Thus, these cases of functionally characterized circRNAs highlight the broad spectrum of physiological processes that are modulated by this new class of regulatory RNAs.

In this study, we identify a collection of circRNAs in cultured *Drosophila* cells in response to bacterial infection. We show that depletion of a brain-enriched circRNA *Edis* results in hyperactivation of antibacterial peptide genes both in cultured cells and *in vivo*. Consequently, flies with reduced levels of *Edis* expression display enhanced clearance of invading bacterial pathogens and heightened resistance to Gram-negative bacterial challenge. Conversely, *Edis* overexpression dampens the innate immunity response. Additionally, flies with whole-body chronic inactivation of *Edis* display shortened lifespan and impaired locomotor activity that further deteriorates with age. Remarkably, depletion of *Edis* exclusively in neurons is sufficient to induce similar hyperactivated immune responses and prominent mobility and longevity defects, coupled with severe phenotypes in neurodevelopment. Lastly, we present evidence that the immune suppressor activity of *Edis* is mediated through a functional protein encoded by this circRNA. Thus our study establishes *Edis* as an essential circRNA encoding a functional protein with key physiological roles in regulating innate immunity and neural development.

## Results

### Identification and validation of circRNAs

We prepared total RNA samples from cultured *Drosophila* S2 cells that were either left untreated or treated with a combination of *20*-*h*ydroxy*e*cdysone (**20-HE**) and a mixture of *Escherichia coli* and *Micrococcus luteus*, which activates innate immunity signaling [6]. To enrich circRNAs, RNA samples were subjected to ribosomal RNA depletion followed by RNase R treatment, which specifically degrades linear RNAs. Subsequently, cDNA libraries were constructed and sequenced using the paired-end strategy. CircRNAs were identified based on the presence of “back-spliced” exon junctions, in which the 3’ end of a downstream exon is linked to the 5’ end of an upstream exon. In total, ~5000 circRNA candidates were identified in various *Drosophila* samples (S1 Table). A significant number of circRNA candidates overlap with those identified in previous studies (S2 Table) [51,54]. Among the 1095 circRNA candidates identified from S2 cells that have been treated with a combination of 20-HE and a mixture of *Escherichia coli* and *Micrococcus luteus*, a significant proportion (37.8%, multi-exonic) contains two or more exons, whereas the majority (58.5%, mono-exonic) is composed of either the whole or partial single annotated exons (S1 Fig and S3 Table). A small fraction (2.1%, labeled as mapping to introns) appears to map exclusively to the intronic region of annotated transcripts. However, they are predominantly antisense to annotated transcripts and likely products of cryptic splicing events, since consensus splice donor and acceptor sequences (5’-GU and 3’-AG) were found to flank the region that gives rise to these circRNAs. The remaining circRNA candidates (1.6%) were collectively labeled as “miscellaneous”. So far, we have not found any intron-containing circRNAs in our validated circRNA collection. These observations suggest that intron-containing circRNAs are not abundant in *Drosophila*. Alternatively, it is possible that the computational strategy employed in this study, which dictates the presence of splice donor and acceptor sites in the flanking region as a prerequisite for circRNA identification, has filtered out intron-containing circRNAs.

To validate circRNAs, we conducted a combination of RT-PCR, Sanger sequencing and RNase R resistance assays. Convergent primer pairs are expected to amplify products from both *g*enomic *DNA* (**gDNA**) and cDNA templates, whereas divergent primer pairs are expected to exclusively amplify regions that encompass the back-spliced exon **junctions** (S2A–S2D Fig). Second, we purified PCR products derived from the divergent primer pairs and performed Sanger sequencing, which confirmed the unique back-spliced exon junctions (S2A and S2B Fig). Third, we performed RNase R treatment, which reveals that circRNAs are resistant, whereas their linear siblings are susceptible to RNase R digestion (S2E and S2F Fig). Thus combined results from RT-PCR, Sanger sequencing and RNase R resistance analyses confirm circular configuration. Altogether, we were able to validate ~50 circRNAs (S4 Table).

### The circRNA *Edis* regulates innate immunity in cultured *Drosophila* cells

**Fig 1:**
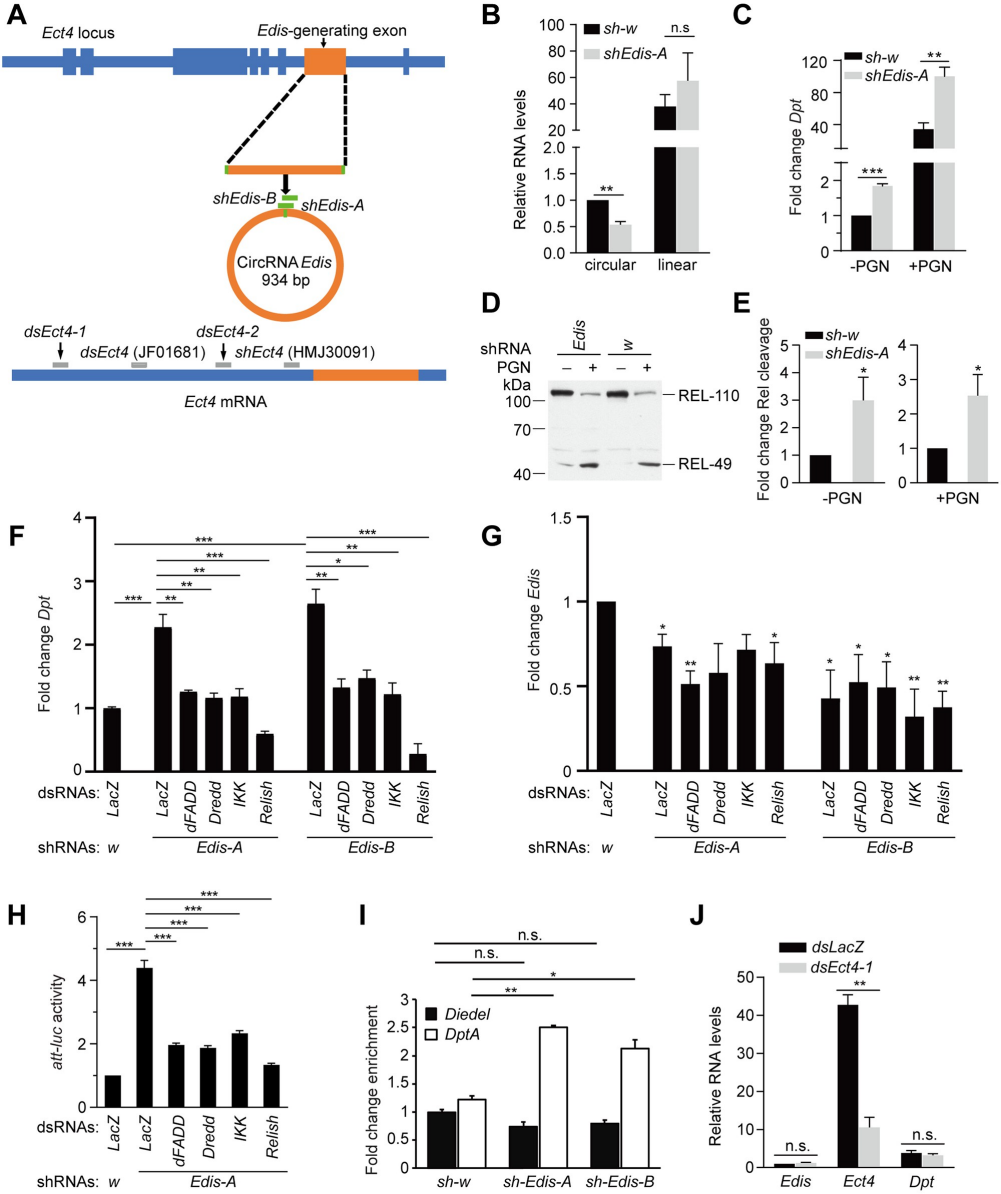
*Edis* depletion impacts innate immunity signaling. (**A**) A diagram depicting the linear *Ect4* mRNA and circular RNA *Edis* derived from the *Ect4* locus as well as the mapping of various dsRNA/shRNA reagents targeting *Ect4* (gray bars) or *Edis* (green bars). Thick and thin bars, respectively, represent exons and introns. (**B-C**) S2 cells stably transfected with an shRNA construct targeting the back-spliced exon junction of *Edis* (*shEdis-A*) or a control (Ctrl) shRNA against *white* (*w*) were treated with 20-hydroxyecdysone (**20-HE**) and subsequently with (+) or without PGN (-). Levels of linear *Ect4* and circular *Edis* transcripts (**B**) or *Diptericin* (***Dpt***) mRNA (**C**) were measured by RT-qPCR and normalized to *RpL32* (student t test, n = 3, * *p*<0.05; ** *p*<0.01; *** *p*<0.001; ns, non-significant). (**D-E**) *Edis* or control knockdown cells were with 20-HE and subsequently with PGN. Relish cleavage was measured by immunoblot (**D**) and quantified (**E**). Right and left panels, respectively, show fold change in Rel cleavage with and without PGN treatment (student t test, n = 3, * *p*<0.05). (**F-G**) S2 cells stably transfected with two independent shRNAs targeting *Edis* (*shEdis-A* or *-B*) or control *sh-w* construct were subsequently transfected with various dsRNAs (below). Cells were treated with 20-HE and levels of *Dpt* mRNA (**F**) and *Edis* (**G**) were measured (student t test, n≥3, * *p*<0.05; ** *p*<0.01; *** *p*<0.001). (**H**) S2 cells stably transfected with the *shEdis* or control shRNA construct, were transfected with the *Att-luc* reporter driven by the antimicrobial peptide gene *Attacin* promoter and the *pActin-Renilla* control reporter constructs together with various dsRNAs against various components of IMD signaling. Cells were treated with 20-HE and reporter gene activities were measured and normalized (student t test, n≥5, *** *p*<0.001). (**I**) S2 cells stably transfected with the *shEdis* or control shRNA construct were transfected with the Flag-Relish and control HA-Ran expression constructs. Cells were treated with copper to induce transgene expression, and subjected to ChIP assay using anti-Flag antibody. Enrichment of DNA fragments from the *DptA* and *Diedel* (control) promoters was measured (one-way ANOVA, n = 2, * *p*<0.05; ** *p*<0.01; ns, non-significant). (**J**) S2 cells were transfected with dsRNA targeting exclusively the linear *Ect4* transcript or a control dsRNA targeting *LacZ*. Cells were treated with 20-HE and levels of *Ect4*, *Edis* and *Dpt* were measured (student t test, n = 3, *** *p*<0.001; ns, non-significant).

To identify circRNAs relevant to innate immunity signaling, we depleted individual circRNAs in S2 cells by designing shRNAs targeting the unique back-spliced exon junctions (Fig 1A). Cells were subsequently treated with 20-HE and then either left untreated or treated with DAP-type *p*eptido*g*lyca*n* (**PGN**), a component of the cell wall of Gram-negative and select Gram-positive bacteria that potently activates IMD antibacterial innate immunity signaling [17]. Next, we performed RT-PCR analysis to examine the impact on levels of the *Diptericin* (***Dpt***) mRNA, which encodes a potent antibacterial peptide. This analysis identified a number of circRNAs that affected *Diptericin* expression (S3 Fig). For subsequent analysis, we focused on *circ_1705* (*circ_Ect4*), which is derived from a single exon of the *Ect4* transcripts (Figs 1A and S4). This circRNA was among the ones identified in previous studies [51,54]. We named it *Edis* (*Ect4*-*d*erived *i*mmune *s*uppressor) based on the phenotypes described below. Levels of *Edis* do not seem to change in S2 cells that are subjected to various combinations of 20-hydroxyecdysone treatment and/or bacterial infection (S5 Fig). Compared to controls, depletion of *Edis* in S2 cells by two independent shRNAs (*shEdis-A and -B*) leads to an increase in *Dpt* mRNA levels, both in the absence and in the presence of PGN treatment (Figs 1A–1C and S6A). In addition, *Edis*-depleted cells display enhanced Relish processing, a hallmark of IMD antibacterial signaling pathway activation (Fig 1D and 1E). Notably, levels of the linear *Ect4* transcript were not affected in *Edis*-depleted cells (Figs 1B and S6B–S6D). Furthermore, the increase in *Dpt* expression elicited by *Edis* knockdown is alleviated by depleting components of the IMD signaling pathway such as dFADD, Dredd, DmIKK (Ird5 and Kenny) or Relish (Fig 1F and 1G). Similar results were obtained by monitoring the expression of a luciferase reporter gene driven by the promoter of the antimicrobial peptide gene *Attacin-A* (*Att*) (Fig 1H). Consistent with these findings, we detected an increase in Relish occupancy on the *Dpt* promoter upon *Edis* depletion, but not on the negative control *Diedel* promoter (Fig 1I), further reinforcing the notion that *Edis* impacts the IMD signaling pathway by regulating Relish activity. In contrast, depletion of linear *Ect4* transcripts by two independent dsRNAs that do not overlap with *Edis* had no significant impact on *Edis* or *Dpt* expression (Figs 1A and 1J, S7). These results demonstrate that the circRNA *Edis* regulates innate immunity in cultured S2 cells.

**Fig 2:**
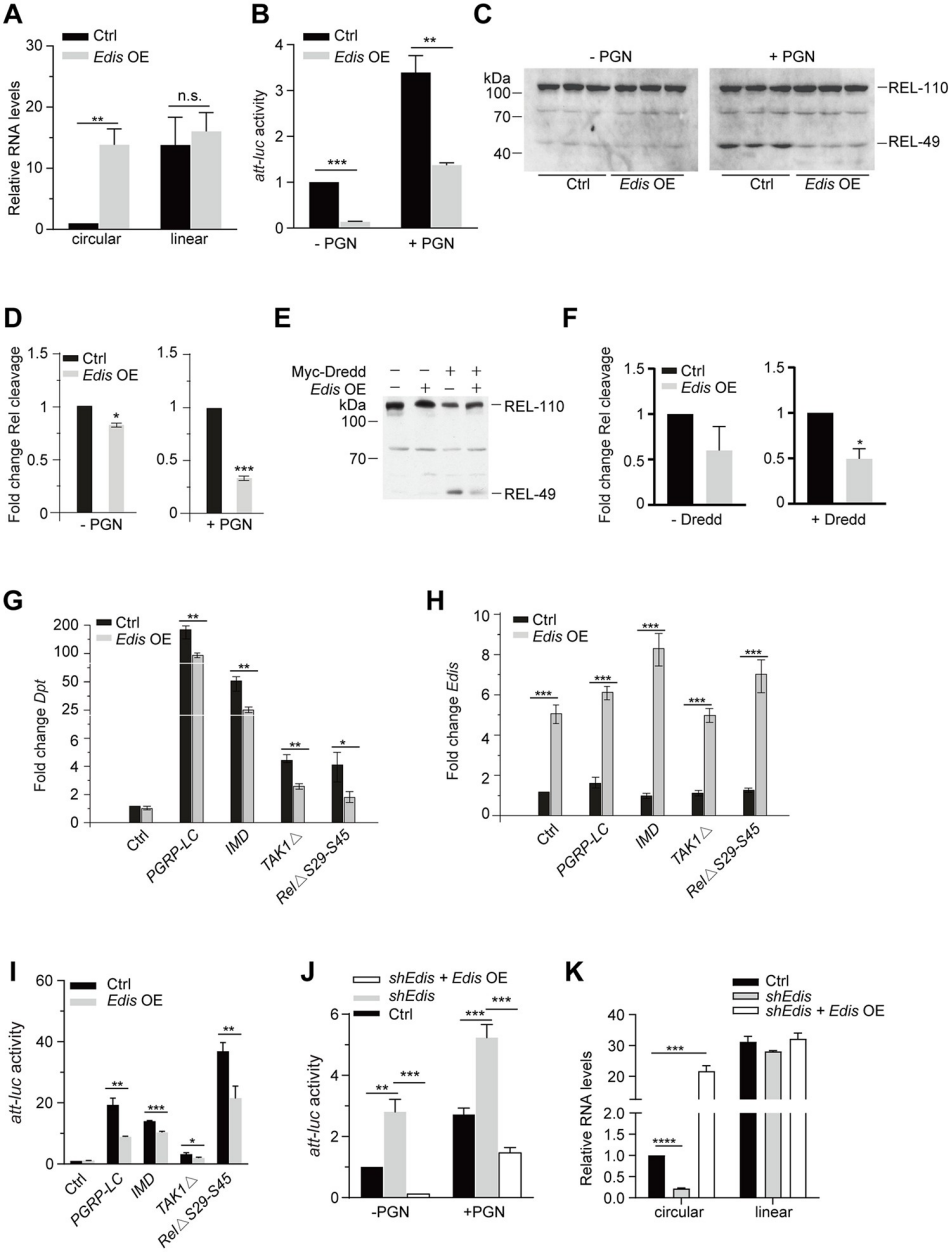
*Edis* overexpression blocks IMD innate immunity signaling. (**A-D**) S2 cells were transfected with an *Edis* expression construct or empty vector, together with the *Att-luc* reporter and the *pActin-Renilla* control reporter. Cells were treated with 20-HE and PGN. Levels of *Ect4* and *Edis* (**A**, student t test, n = 3, *** *p*<0.001; ns, non-significant), normalized reporter activity (**B**, student t test, n = 3, ** *p*<0.01; *** *p*<0.001), and Relish processing (**C**) are shown. Quantification of Relish processing immunoblot in **C** is shown in **D** (student t test, n = 3, * *p*<0.05; *** *p*<0.001). (**E-F**) A stable S2 cell line expressing Myc-tagged Dredd under the control of the copper-inducible *metallothionein* promoter was transfected with either the expression vector for *Edis* or the control empty vector. Cells were first treated with and 20-HE. Subsequently, cells were treated with copper to induce transgene expression, or left untreated. Relish processing was measured by immunoblot (a representative image of three independent experiments is shown). Quantification of Relish immunoblot is shown in **F** (student t test, n = 3, * *p*<0.05). (**G-H**) S2 cells were transfected with various combinations of expression constructs for IMD signaling components (below), together with *Edis* overexpression or empty vectors. Cells were treated with 20-HE and levels of *Dpt* mRNA (**G**) and *Edis* (**H**) were measured (student t test, n = 4, * *p*<0.05; ** *p*<0.01; *** *p*<0.001). (**I**) S2 cells were transfected with various combinations of expression constructs for IMD signaling components (below), together with *Edis* overexpression or empty vectors and the *Att-luc* reporter and the *pActin-Renilla* control reporter constructs. Cells were treated with CuSO_4_ and 20-HE and reporter activity was measured and shown (student t test, n≥4, * *p*<0.05; ** *p*<0.01; *** *p*<0.001). (**J-K**) Various combinations of *Edis* overexpression, shRNA and empty vectors, together with the *Att-luc* reporter and the *pActin-Renilla* control reporter constructs were transfected into S2 cells. Cells were first treated with CuSO_4_, and subsequently with 20-HE and PGN. Reporter activity (**J**, student t test, n = 6, * *p*<0.05; ** *p*<0.01; *** *p*<0.001) and levels of *Ect4* and *Edis* (**K**, student t test, n = 3, *** *p*<0.001; **** *p*<0.0001) were measured. Note that the ectopically expressed *Edis* harbors a back-spliced exon junction that is distinct from that in its endogenous counterpart, therefore, is resistant to shRNA-mediated knockdown.

Next, we examined the impact of *Edis* overexpressing on the IMD pathway in S2 cells [62]. Overexpression of *Edis*, but not a control circRNA *circ_714*, causes a strong reduction in the activity of the *Att-luc* reporter and/or *Dpt* mRNA levels both in the presence and in the absence of PGN treatment (Figs 2A and 2B, S8). In addition, Relish cleavage, elicited by either PGN treatment (Fig 2C and 2D) or ectopic expression of Dredd (Fig 2E and 2F), the caspase that carries out Relish endoproteolytic processing, is significantly compromised by *Edis* overexpression. Furthermore, we found that *Edis* overexpression mitigates the activation of both *Dpt* expression (Fig 2G and 2H) and the *Att-luc* reporter gene (Fig 2I) by ectopic expression of select components of IMD signaling (full length PGRP-LC or IMD, or constitutively active forms of dTAK1 (TAK1Δ) or Relish (RelΔS29-S45)) [22,23]. Lastly, upon restoration of *Edis* expression in *Edis*-knockdown cells, the AMP hyperactivation phenotype is suppressed (Fig 2J and 2K). Note that the exogenous *Edis* product from the circRNA expression construct contains back-spliced exon junction sequences derived from the vector that are distinct from those in the endogenous *Edis*. Therefore, the exogenous *Edis* is shRNA-resistant. Importantly, the linear *Ect4* transcript levels remain unchanged (Fig 2A and 2K). We conclude that the circRNA *Edis* suppresses innate immunity signaling in cultured S2 cells.

### *Edis* regulates innate immunity *in vivo*

**Fig 3:**
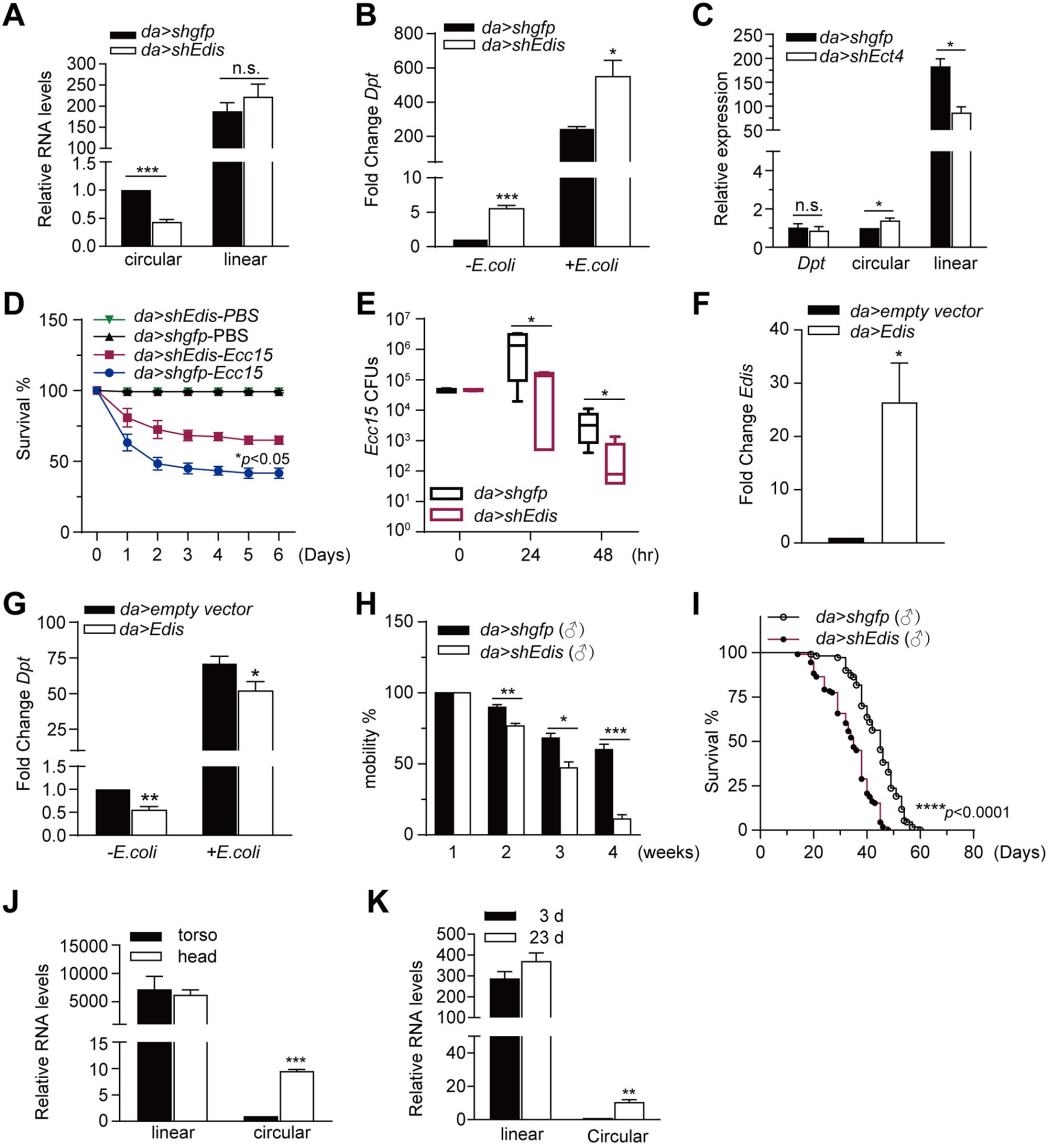
*Edis* regulates innate immunity *in vivo*. (**A-B**) Flies carrying the ubiquitously expressed *daughterless-Gal4* driver and a temperature-sensitive *Gal80* were crossed to *UAS-shEdis* or control *shGFP* flies. Fly crosses were kept at 18°C. Adult progeny were shifted to 29°C for 5 days to allow for shRNA transgene expression. Flies were either left untreated or infected by *E*. *coli*, total RNAs were extracted 6-h post-infection and levels of linear *Ect4* and circular *Edis* RNA (**A**) and *Dpt* (**B**) were measured (student t test, n≥3, * *p*<0.05; *** *p*<0.001; ns, non-significant). (**C**) The *UAS-shEct4* was employed to knockdown the linear *Ect4* RNA *in vivo* using a similar strategy as in **A**. Levels of *Ect4*, *Edis* and *Dpt* were measured (student t test, n = 3, ** *p*<0.01; ns, non-significant). (**D**) *Edis* knockdown and control flies in multiple groups (20–25 flies per group) were injected with a concentrated culture of *Erwinia carotovora carotovora 15* (*Ecc15*) or PBS (control). Fly survival was recorded daily and plotted (log rank test, n≥3, * *p*<0.05). (**E**) At various time points post *Ecc15* infection, groups of 4 flies were homogenized in sterile PBS. Fly homogenates were diluted and plated onto Ampicillin-LB plates, and the resultant colonies were counted. Shown are *c*olony-forming *u*nits (**CFU**s) per fly (student t test, n≥4, * *p*<0.05). (**F-G**) The *UAS-Edis* transgenic line was employed to overexpress *Edis in vivo* using a similar strategy as in **A**. The *UAS-laccase 2 empty vector* transgene was included in the crossing scheme as a control. Flies were either left untreated or infected by *E*. *coli*, total RNAs were extracted 6-h post-infection, and levels of *Edis* (**F**, student t test, n = 3, * *p*<0.05**)** and *Dpt* (**G**, student t test, n = 6, * *p*<0.05; ** *p*<0.01) transcripts were measured. (**H**) Male *da>shEdis; Gal80*^*ts*^ flies and control *da>shGFP; Gal80*^*ts*^ flies were kept at 29°C for indicated period of time (below). Flies in groups of 15 were placed into conical culture tubes and tapped to the bottom, and the percentage of flies that can climb over the 2-centimeter mark within 15 seconds was recorded and shown (student t test, n≥4, * *p*<0.05; ** *p*<0.01; *** *p*<0.001). (**I**) Male *da>shEdis; Gal80*^*ts*^ flies and control *da>shGFP; Gal80*^*ts*^ flies in multiple groups of 25 were kept at 29°C. Fly survival was recorded daily and plotted (log rank test, n≥6, **** *p*<0.0001). (**J**) Total RNA was prepared from either fly head or torso samples, and levels of linear *Ect4* and circular *Edis* RNA were measured (student t test, n = 3, *** *p*<0.001). (**K**) Levels of linear *Ect4* and circular *Edis* transcripts in RNA samples from heads of young (3d) and old (23d) flies were measured (student t test, n = 3, ** *p*<0.01).

To further assess the role of *Edis in vivo*, we generated shRNA transgenic lines targeting *Edis* [63] and silenced *Edis* expression by crossing *shEdis* animals to ubiquitously expressed driver lines, including the medium strength *daughterless-Gal4* (***da-Gal4***), and the strong *Actin5C-Gal4* and *Tubulin-Gal4* drivers. Global depletion of *Edis* by *Actin5C-Gal4* or *Tubulin-Gal4* causes lethality, whereas *Edis* knockdown by *da-Gal4* yields a low number of progeny flies. To bypass the developmental lethality associated with *Edis* knockdown, we employed the *da-Gal4; tub-Gal80*^*ts*^ composite line [43]. At the permissive temperature (18°C), Gal80 inhibits Gal4 and shuts off the shRNA transgene; whereas at the restrictive temperature (29°C), Gal80 is inactivated, thereby allowing for the expression of the shRNA transgene. Fly crosses were kept at 18°C. Upon eclosion, age-matched progeny of appropriate genotypes was shifted to and maintained at 29°C for 5–7 days to allow for shRNA transgene expression and target gene silencing. This strategy allowed us to achieve reproducible knockdown of *Edis* in adults, while levels of the linear *Ect4* transcript remain unaltered (Fig 3A). A control cross was set up using a shRNA transgenic line targeting *gfp*. Consistent with a recent report, we detected higher levels of *Edis* in flies kept at 29°C than those at 18°C (S9 Fig) [51]. Compared with controls (*tub-Gal80*^*ts*^*; da>shGFP*), depletion of *Edis in vivo* leads to markedly elevated levels of the *Dpt* transcript under both non-infection and *E*. *coli* infection conditions (Fig 3B). This is consistent with the phenotype observed in S2 cells (Figs 1B and 1C, S6A). Next, we employed a similar strategy to deplete the linear *Ect4* transcript using an shRNA transgene and a long hairpin dsRNA transgene that exclusively target the linear *Ect4*, but *Edis* (Fig 1A). We found that global *Dpt* gene expression upon *Ect4* knockdown remains comparable to that in controls (Figs 3C and S10). These results demonstrate that the circRNA *Edis* impacts innate immunity *in vivo*.

To further define the functional relevance of *Edis* in innate immunity, we injected either sterile PBS or concentrated culture of the fly pathogen *Erwinia carotovora carotovora* strain 15 (*Ecc15*) into *Edis*-depleted or control animals and monitored fly survival. While the survival of all flies displayed a decrease following *Ecc15* infection, *Edis*-depleted animals present a significantly higher survival rate than controls (Fig 3D) that correlates with AMP expression levels. To further examine whether enhanced survival is attributable to improved pathogen clearance or immune tolerance, we monitored pathogen load at various time points post-infection. This analysis revealed that *Edis*-depleted animals out-performed controls in clearing *Ecc15* (Fig 3E). We conclude that *Edis*-depletion leads to enhanced antibacterial defense *in vivo*.

Next, we examined the impact of ectopic *Edis* expression on innate immunity in adult flies using by crossing *da-Gal4; tub-Gal80*^*ts*^ flies to *UAS-Edis* or *UAS-empty vector* (control) animals. We found that *Edis* overexpression leads to a reduction in levels of the *Dpt* transcript under both non-infection and *E*. *coli* infection conditions (Fig 3F and 3G). Taken together, these observations solidify the notion that *Edis* plays an important role in suppressing innate immunity *in vivo*.

### Neuronal *Edis* impacts innate immunity and animal survival

We noticed that *da>shEdis* animals show progressively impaired locomotor activity over time as compared to age-matched controls (Figs 3H and S11A) and a shorter lifespan (Figs 3I and S11B). To further investigate the physiological basis of this phenotype, we examined *Edis* expression in different tissues (S12A Fig). We also detected 9-fold higher levels of *Edis* in the fly head than in the torso (Fig 3J). In addition, our analysis reveals that in fly heads, levels of *Edis*, but not its linear sibling *Ect4*, display a dramatic increase with age (Fig 3K). As circRNAs are generally more stable than their linear siblings, the apparent increase in levels of circRNA over age could be due, at least in part, to differences in stability of circular and linear RNAs. These findings are consistent with recent reports showing that select circRNAs are enriched in the nervous system and further regulated by age [51,54].

**Fig 4:**
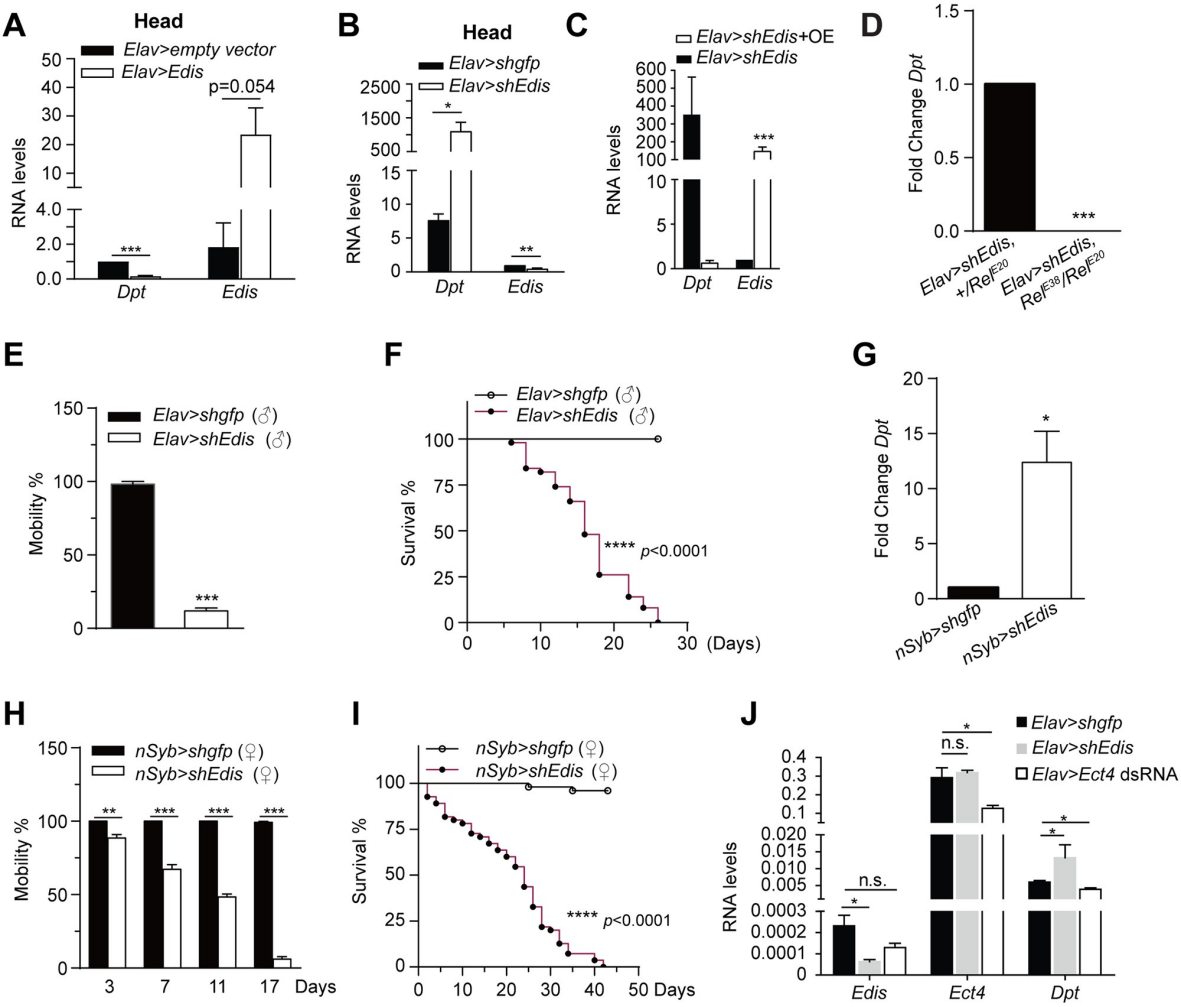
Neuron-specific *Edis* depletion leads to hyperactivation of innate immunity, impaired locomotion and shortened lifespan. (**A**) The *UAS-Edis* or control *UAS-empty vector* transgenic flies were crossed to the *Elav-Gal4* driver line. Total RNA was prepared from dissected fly heads, and levels of the circular *Edis* RNA or *Dpt* mRNA were measured (student t test, n = 3, * *p*<0.05; *** *p*<0.001). (**B**) The *UAS-shEdis* or control *UAS-shGFP* flies were crossed to the neuron-specific *Elav-Gal4* driver line. Levels of the *Ect4*, *Edis* or *Dpt* in fly head samples were measured (student t test, n = 3, * *p*<0.05; *** *p*<0.001). (**C**) The *UAS-Edis* or control *UAS-laccase 2 empty vector* transgenes were introduced into the *Elav>shEdis* flies. Total RNA was prepared from dissected fly heads, and levels of the circular *Edis* RNA or *Dpt* mRNA were measured (student t test, n = 3, *** *p*<0.001). (**D**) The *UAS-shEdis* flies were crossed to the *Elav-Gal4* driver line. Female progeny either in the *Relish* heterozygous (*Rel*^*E20*^/+) or trans-heterozygous (*Rel*^*E20*^/*Rel*^*E38*^) background were collected at ~3 weeks of age, and levels of *Dpt* in fly heads were measured (student t test, n = 3, *** *p*<0.001). (**E**) Locomotor activity of 2-5d old Male *Elav>shEdis* and control flies were measured and shown (student t test, n≥4, *** *p*<0.001). (**F**) Survival of male *Elav>shEdis* and control flies were measured and shown (log rank test, n = 4 to 8, **** *p*<0.0001). (**G-I**) The *UAS-shEdis* or control *UAS-shGFP* flies were crossed to a second neuron-specific *nSyb-Gal4* driver line. Level of *Dpt* RNA in fly heads (**G**, student t test, n = 3, * *p*<0.05), climbing activity (**H**, student t test, n≥3, *** *p*<0.001) and lifespan (**I**, log rank test, n = 4 to 11, **** *p*<0.0001) of female progeny are shown. (**J**) The *UAS-dsEct4*, *UAS-shEdis* or control *UAS-shGFP* flies were crossed to the neuron-specific *Elav-Gal4 UAS-Dcr-2* driver line. Levels of *Ect4*, *Edis* and *Dpt* transcripts were measured (student t test, n = 3, n = 2 for *Elav>shEdis* samples for measuring *Dpt* levels, * *p*<0.05; ns, non-significant).

To further explore the functional importance of neuron-derived *Edis*, we first overexpressed *Edis* in neurons using the pan-neuronal *Elav-Gal4* driver (*c155*) and found it potently blocked *Dpt* expression (Fig 4A). Next, we depleted *Edis* in neurons by crossing *Elav-Gal4* and *shEdis* flies. Remarkably, neuronal *Edis* knockdown by two independent shRNAs is sufficient to cause elevated levels of a panel of AMP transcripts in RNA samples prepared from fly heads (Figs 4B and S13A). Importantly, the immunity phenotype in *Elav>shEdis* flies was rescued by restoring *Edis* expression in neurons (Fig 4C), and increased *Dpt* gene expression is blocked in *Relish* null mutants (*Rel*^*E20*^/*Rel*^*E38*^) compared with *Relish* heterozygous animals (*Rel*^*E20*^/+) (Fig 4D). This is consistent with the observation in S2 cells that elevated AMP gene expression upon *Edis* knockdown depends on Relish (Fig 1F–1H). Moreover, flies with neuronal *Edis* depletion similarly display significantly impaired locomotor activity that deteriorates rapidly with age. Notably, male *Elav-Gal4>shEdis* flies already show severe locomotor activity defects at a young age (2-5d) (Fig 4E), much earlier than that observed in female counterparts (~14d, S11C Fig), possibly due to dosage-compensation of X-linked *Elav-Gal4* and/or differences in both *Edis* levels and knockdown efficiency in males and females (S14 Fig). Furthermore, *Elav>shEdis* flies display a shortened lifespan compared to controls (Figs 4F and S11D). To confirm these findings, we crossed *shEdis* flies to a second neuron-specific driver, *nSyb-Gal4*. We found that the *nSyb>shEdis* animals phenocopy *Elav>shEdis* flies (Fig 4G–4I). In contrast, glia-specific depletion of *Edis* does not impact innate immunity gene expression (S12B Fig). It is currently unclear whether glia-specific depletion of *Edis* impacts animal behavior or survival.

Consistent with the report that *Ect4* mutants display an increase in AMP gene expression in the tracheal airway epithelium [64], trachea-specific depletion of *Ect4* led to elevated *Drosomycin-GFP* reporter activity (S15 Fig). To examine the potential role of *Ect4* in neurons, we depleted *Ect4* using the *Elav-Gal4* driver. We actually detected a decrease in *Dpt* mRNA levels in *Ect4*-depleted fly heads, whereas the opposite phenotype was observed upon *Edis* depletion (Figs 4J and S16). The opposing roles of *Ect4* in innate immunity in different cell types (neurons and trachea) may explain the lack of significant changes in global AMP gene expression in RNA samples prepared from whole flies with ubiquitous *Ect4* depletion (Figs 3C and S10). Taken together, these results show an essential and potent role of neuron-derived *Edis* in regulating innate immunity and animal survival.

### *Edis* is essential for neural development

**Fig 5:**
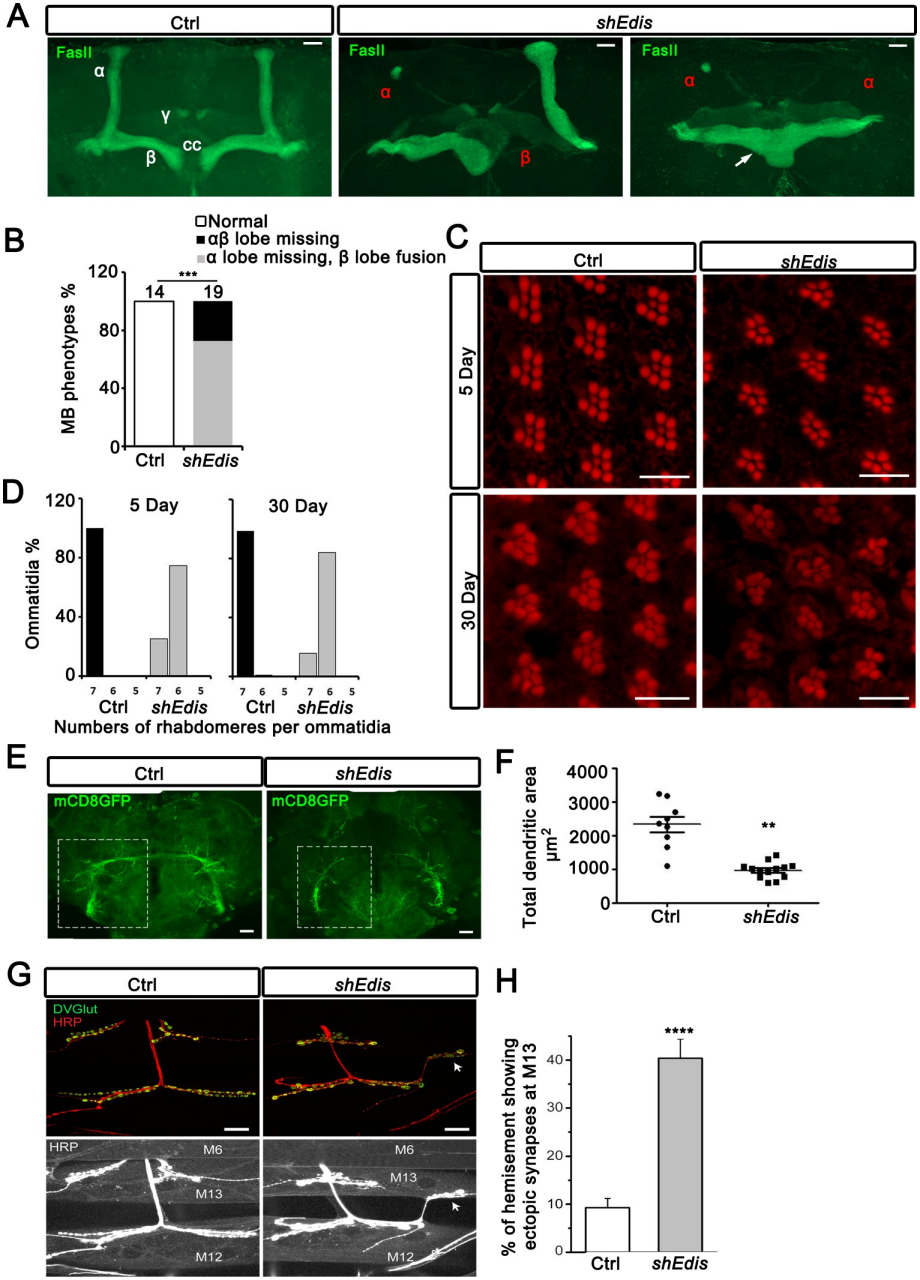
Neuron-specific depletion of *Edis* causes abnormal axonogenesis and neurodevelopment. **(A)** Confocal images of dorsal anterior regions of adult brains with neuron-specific expression of control *shgfp* (left panel) or *shEdis* (middle and right panels) driven by *Elav-Gal4* driver. In control brain (left panel), anti-FasII antibody delineates the central complex (CC) as well as the vertical α and horizontal β and γ lobes of the mushroom bodies (**MB**s), with γ lobes showing weaker FasII signal, as indicated. Depletion of *Edis* resulted in a spectrum of severe, age-dependent morphological defects in the MBs. In middle panel, one α lobe and one β lobe were mostly missing, and the remaining α and β lobes showed abnormal morphology. In right panel, both α lobes were largely missing, while the two β lobes crossed the midline and merged together. Scale bar: 20 μm. **(B)** Quantification of MB morphology phenotypes shown in **A** (Chi-squared test, sample numbers are shown on top, *** *p*<0.001). (**C**) To deplete *Edis* in the eye, *GMR-Gal4* was used to drive *shEdis* transgene expression. The adult eyes of Ctrl and *GMR>shEdis* flies were analyzed by phalloidin staining (red) at day 5 or day 30 after eclosion. Scale bar: 10 μm. (**D**) Images of the 5-day- or 30-day-old fly eyes with the indicated genotypes were quantified and shown as the percentage of ommatidia with the indicated numbers of rhabdomeres per ommatidia (n = 3–5). (**E**) To probe the role of *Edis* in the giant fiber system, *ok307-Gal4* was used to drive *shEdis* transgene expression, the giant fiber system was revealed using a membrane-bound *mCD8-eGFP* reporter (green). The dendritic area of giant fiber system was highlighted with dotted boxes. Scale bar: 20 μm. (**F**) Quantification of the average total dendritic area covered by giant fiber system dendritic termini (student t test, n = 9–13; ****, *p*<0.0001). (**G**) Representative confocal images of muscle 12 (M12) and muscle 13 (M13) NMJs of control (*Elav>shGFP*) and *Edis* knockdown (*Elav>shEdis*) 3rd instar larvae. The top panels show nerve terminal (anti-HRP) and synaptic vesicles (anti-DVGlut), while the bottom panels show over-saturated HRP signals for the same image to highlight muscle 6, 12 and 13. Arrow indicates an ectopic synapse formed at M13 as an extension from the M12 NMJ in *Elav>shEdis* animals. Scale bar = 20 μm. (**H**) Quantification of percentage of hemisegments in control and *Edis* knockdown larvae that show ectopic synapses at muscle 13 (student t test, ~10 hemisegments from each animal were scored. Nine larvae from each genotype were analyzed, **** *p*<0.0001).

Given the strong mobility and viability phenotypes associated with neuronal *Edis* depletion, we next investigated its effect on brain anatomy. Brains from young (4-6d) *Elav>shEdis* males or controls were dissected and stained with anti-Fasciclin II (FasII) antibodies, which highlight the *m*ushroom *b*odies (**MB**s), a distinct brain structure involved in learning and memory [65–70]. Notably, in young fly brains depleted of *Edis*, MBs display a spectrum of severe morphological defects (missing, split and merged MB lobes) (Fig 5A and 5B). In addition, depletion of *Edis* in the eye using *GMR-Gal4* leads to defective ommatidium structure, which further deteriorates with age (Fig 5C and 5D). Furthermore, as giant fiber is another neuronal structure with stereotypical morphology that is easy to visualize, we examined the impact of *Edis* depletion on grant fiber morphology. We found that *Edis* depletion in a subset of neurons using *ok307-Gal4* results in abnormal giant fiber morphology and a decrease in dendritic area (Fig 5E and 5F). Moreover, *Elav-shEdis* larvae show substantial wiring defects of the neuromuscular junctions (NMJs)—a significantly higher frequency of ectopic synaptic formation from muscle 12 to muscle 13 comparing with control larvae (Fig 5G and 5H). We did not detect any significant changes in bouton numbers in the muscles.

**Fig 7:**
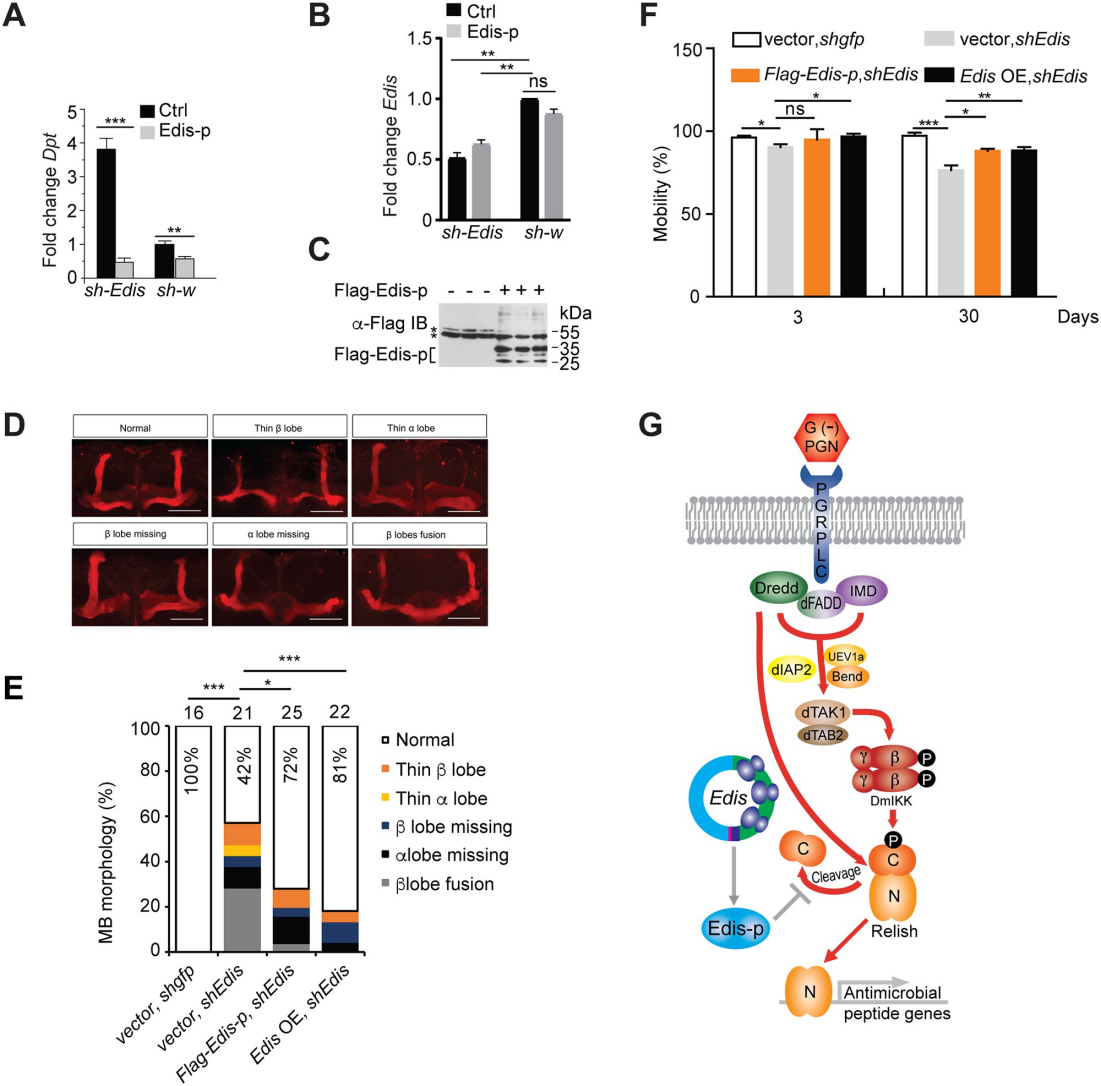
Edis-p rescues the innate immunity and neurodevelopment phenotypes elicited by *Edis* depletion. (**A-C**) Stably transfected S2 cells carrying the pMT-Gal4 and *UAS-shEdis* or *UAS-sh-w* (control) constructs were transfected with empty vector or an expression construct encoding sh*Edis*-resistant Flag-tagged Edis-p. Cells were either left untreated or treated with PGN and levels of the *Dpt* (**A**) and *Edis* (**B**) transcripts were measured by RT-qPCR (student t test, n = 3, ** *p*<0.01; *** *p*<0.001; ns, non-significant). (**C**) An anti-Flag immunoblot shows Fag-Edis-p expression in samples from **A**. Non-specific bands are labeled with asterisks. (**D**) Confocal images of dorsal anterior regions of adult brains with various MB morphology phenotypes. Depletion of *Edis* in neurons resulted in a spectrum of severe, age-dependent morphological defects in the MBs as noted above each panel. (**E**) MB morphology phenotypes were quantified in 3-5d old flies with neuron-specific expression of control *shgfp* or *shEdis*, together with an *UAS-Edis*, *UAS-Flag-Edis-p* or empty vector transgene, driven by the *Elav-Gal4* driver (Chi-squared test). Sample numbers and percentage of normal MB in each genotype are shown (* *p*<0.05; *** *p*<0.001). **(F)** Climbing activity of 3d- and 30d-old flies of the same genotype as in **E** is shown (student t test, n = 2–5, * *p*<0.05; ** *p*<0.01; *** *p*<0.001; ns, non-significant). (**G**) A model of the proposed mechanism of function of *Edis* in the IMD antibacterial innate immunity signaling pathway.

Since *Ect4* and *Edis* arise from the same gene locus, they carry identical nucleotide sequences except for the back-spliced exon junction that is unique to *Edis*. Thus it is of high importance to ensure the specificity of the RNAi reagents employed in our study. To this end, we have employed independent shRNA/dsRNA reagents that specifically target *Ect4* or *Edis* (Fig 1A). It is unlikely that the innate immunity and neurodevelopment phenotypes in *Edis* knockdown animals are due to off-target effects, because 1) Two independent shRNA transgenes against *Edis* generate similar phenotypes both *in vivo* and in cultured S2 cells (Figs 1B, 1C, 4B, 5A and 5B, S6A and S13); 2) restoring *Edis* expression in neurons rescues both the innate immunity hyperactivation and MB morphology phenotypes of *Elav-shEdis* flies (Figs 4C, 7E and 7F); 3) our shRNA constructs against *Edis* do not cause significant off-target effects (S5 Table and S6B–S6D Fig), and do not impact *Ect4* mRNA levels (Figs 1B, 2K, 3A and 4J, S6B, S6C, S16A and S16B); and 4) while due to lack of anti-Ect4 antibodies we could not assess the potential impact of *Edis* depletion on translation of the endogenous *Ect4* mRNA, we found that *Edis* depletion did not affect the production or stability of Flag-Ect4 (S17 Fig).

*Ect4* mutants display severe defects in neuron development, morphology and cell death upon injury [71]. In our hands, flies with neuronal *Ect4* depletion (*Elav>shEct4* and *Elav>dsEct4*) do not display defects in either mobility or MB morphology. We note the moderate *Ect4* knockdown efficiency in these flies (Figs 4J and S16C–S16E). It is possible that the surviving *Ect4* knockdown animals are “escapers” with a moderate degree of *Ect4* depletion, as more efficient knockdown may cause lethality. Nonetheless, we detected a consistent decrease in *Dpt* RNA levels in both *Elav>shEct4* and *Elav>dsEct4* animals, which is opposite to the phenotype of *Elav>shEdis* animals (Figs 4J and S16C–S16E). These results suggest that *Ect4* and *Edis* seem to positively and negatively, respectively, regulate innate immunity gene expression in neurons. On the other hand, we note that trachea-specific depletion of *Ect4* led to elevated expression of AMP genes (S15 Fig), suggesting that *Ect4* represses innate immunity gene expression in the trachea. These observations are consistent with the phenotypes previously reported for *Ect4* mutants [64]. Thus our experimental settings allowed us to achieve a functionally relevant extent of *Ect4* knockdown in the trachea. It is unclear whether *Edis* impacts AMP gene expression in the trachea. We conclude that the immunity and neurodevelopmental phenotypes of *Elav>shEdis* animals are not due to off target effects (by affecting *Ect4* expression), and that *Ect4* appears to differentially regulate AMP gene expression in neurons and trachea. It has been suggested that the *Tollo*/*DNT1*/*Ect4* axis negatively impacts AMP gene expression in trachea [64]. The mechanism underlying the role of *Ect4* in regulating AMP gene expression in neurons remains to be elucidated.

### *Edis* encodes a functional protein

**Fig 6:**
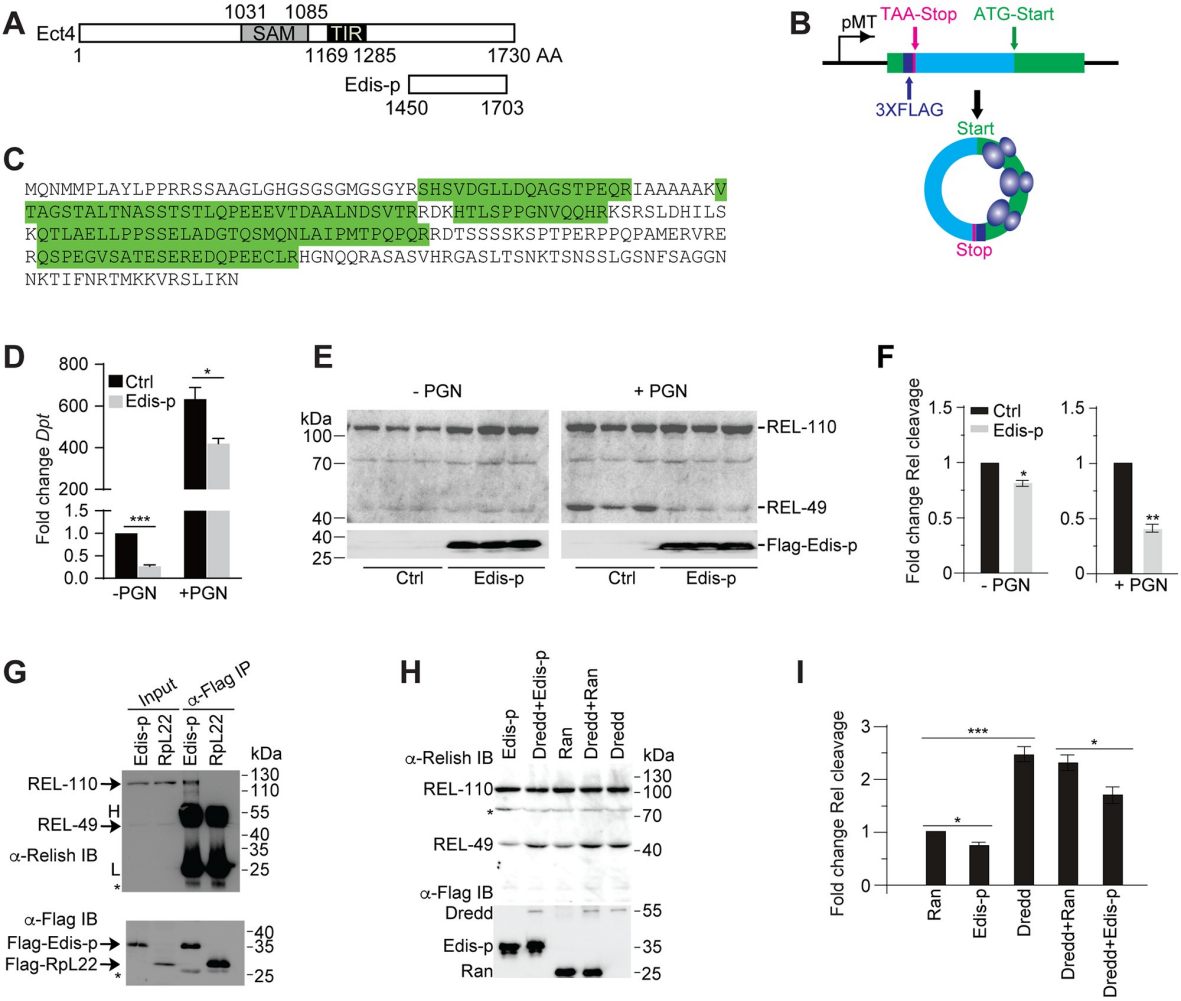
*Edis* encodes a functional protein. (**A**) Schematic of the Ect4 and Edis-p proteins. Positions of the SAM (sterile alpha motif) and TIR (Toll/interleukin-1 receptor homology) domains are shown. (**B**) Schematic of the *Edis* minigene driven by the *metallothionein* promoter. The longest ORF, which encodes a potential protein product (Edis-p) derived from *Edis*, and the corresponding UTR are shown in green and blue, respectively. Positions of the initiation codon and stop codon are noted. A 3X FLAG epitope tag-coding sequence was placed immediately upstream of the putative stop codon. (**C**) Lysate from stably transfected S2 cells carrying the constructs described in **B** was subjected to anti-FLAG IP followed by mass spectrometry. Five independent peptides (in green) derived from the Edis-p were identified. (**D**) S2 cells were transfected with empty vector or the expression construct encoding Flag-tagged Edis-p. Cells were first treated with copper and 20-HE. Subsequently, cells were treated with PGN or left untreated. Levels of the *Dpt* transcript were measured by RT-qPCR (student t test, n = 3, * *p*<0.05; *** *p*<0.001). (**E-F**) S2 cells were transfected with empty vector or the expression construct encoding Flag-tagged Edis-p. Cells were first treated with copper and 20-HE. Subsequently, cells were treated with PGN or left untreated. Cell lysates were subject to immunoblot using anti-Relish (upper panel) or anti-Flag (lower panel) antibodies. Quantification of Relish immunoblot in **E** is shown in **F** (student t test, n = 3, * *p*<0.05; ** *p*<0.01). (**G**) Lysates from stably transfected S2 cells expressing either Flag-Edis-p or Flag-RpL22 (control) were subjected to anti-Flag immunoprecipitation. Subsequently, the immune-purified anti-Flag samples, together with input lysates, were analyzed by immunoblot in parallel using antibodies against Relish (upper panel) and Flag (lower panel). Non-specific bands (asterisks), heavy (H) and light (L) chains are also labeled. (**H**) S2 cell lysate (containing endogenous Dredd and Relish) was incubated with various combinations of purified recombinant Flag-Edis, Flag-Ran and Flag-Dredd in a Relish processing reaction at 30°C for 2h. The reactions were subsequently subjected to immunoblot analysis using anti-Relish and anti-Flag antibodies. (**I**) Quantification of Relish processing in **H** (student t test, n = 3, * *p*<0.05; *** *p*<0.001).

Select circRNAs can be translated into functional proteins [59,60]. We therefore explored the protein-coding potential of *Edis*. *Edis* harbors several potential *o*pening *r*eading *f*rames (**ORF**s). The longest one spans across and terminates immediately downstream of the back-spliced exon junction. It potentially encodes a protein of 254 amino acids (Edis-p), which overlaps with the C-terminus of the Ect4 protein (Fig 6A). Next, we expressed in S2 cells an *Edis* minigene that contains a 3XFlag epitope tag immediately upstream of the stop codon (Fig 6B). Mass spectrometry analysis of immune-purified anti-Flag complex identified five independent peptides derived from Edis-p, thereby demonstrating the protein-coding potential of *Edis* (Fig 6C). We conclude that *Edis* can encode a protein, at least in the transgene setting.

Next, we expressed Flag-tagged Edis-p in S2 cells and examined the impact on innate immunity. We found that ectopic expression of Edis-p phenocopies *Edis* overexpression, as it blocks PGN-induced *Dpt* gene activation (Fig 6D) and Relish cleavage (Fig 6E and 6F). In addition, we detected endogenous Relish in immune-purified Flag-Edis-p, but not the control Flag-RpL22 complexes (Fig 6G), suggesting that Edis-p interacts (either directly or indirectly) with Relish. This is further supported by the co-localization between epitope-tagged Relish and Edis-p in S2 cells (S18 Fig). However, epitope-tagged Relish does not co-immunoprecipitate with Edis-p. It is possible that the Relish-Edis-p interaction is unstable. Alternatively, the epitope tag on Relish could hinder such interaction. Nonetheless, given these observations, the simplest explanation is that Edis-p blocks Relish processing by sequestering Relish. This is consistent with our findings that the AMP gene hyperactivation phenotype elicited by *Edis* knockdown is suppressed by depletion of dFADD, IKK, and Dredd (Fig 1F–1H), and that *Edis* blocks innate immunity activation caused by ectopic expression of PGRP-LC, IMD or TAK1Δ (Fig 2G–2I), as all these IMD pathway components play key roles in Relish activation. In line with this notion, we found that purified recombinant Flag-Edis-p, but not the control protein Flag-Ran, compromises *in vitro* Relish processing catalyzed by both endogenous and exogenous Dredd (Fig 6H and 6I) [72]. Importantly, Edis-p suppresses the AMP gene hyperactivation phenotype elicited by *Edis* depletion in cultured S2 cells (Fig 7A–7C).

Next, we examined whether Edis-p can rescue the neurodevelopmental defects in *Edis*-depleted animals. We found that restoring *Edis* expression in *Elav>shEdis* neurons significantly rescued the MB neurodevelopmental defects. Specifically, the percentage of brains showing normal MB morphology dramatically increased to 81% upon restoring *Edis* levels, whereas only 42% of *Elav>shEdis* brains display normal MB morphology (Fig 7D and 7E). Importantly, the proportion of brains showing normal MB morphology was enhanced to 72% upon Edis-p expression (Fig 7E). Similarly, defects in mobility (Fig 7F) were also rescued by expressing *Edis* or Edis-p. We note that while Edis-p can rescue the MB neurodevelopmental defects in *Edis*-depleted neurons, the extent of rescue is milder than that of the circular RNA form of *Edis* (Fig 7E). Nonetheless, these observations suggest that during neurodevelopment, *Edis* functions at least in part, via the encoded protein Edis-p. However, we cannot exclude the possibility that *Edis* may also operate through other mechanisms. We conclude that *Edis* encodes a functional protein that suppresses innate immunity and regulates neurodevelopment by compromising Relish processing.

## Discussion

In this study, we identify and validate circRNAs in cultured *Drosophila* cells in response to bacterial infection. We show that depletion of the circRNA *Edis* causes hyperactivation of the IMD antibacterial innate immunity signaling pathway both in cultured cells and *in vivo*. Conversely, ectopic *Edis* expression dampens innate immunity activation. Furthermore, silencing of *Edis* either in the whole organism or specifically in neurons leads to constitutive activation of the IMD pathway, accompanied by dramatic climbing deficits and shortened lifespan, which correlate with strong defects in brain structure formation. Lastly, we provide evidence that *Edis* encodes a functional protein that suppresses innate immunity signaling. Thus our study establishes *Edis* as a key regulator of both basal and bacterial infection-stimulated immunity signaling, and a modulator of neural development, potentially serving as an integrator of immune response and neuronal development.

Our study reveals that while *Edis* and *Ect4* are derived from the same parental gene, the circular/linear ratio varies spatiotemporally (Figs 3J and 3K, S12A). In addition, *Edis* and *Ect4* operate in a distinct yet overlapping set of cell/tissue types: 1) Both *Ect4* and *Edis* are critically required for neural cell physiology. *Ect4* is crucial for axon remodeling and Wallerian degeneration in response to injury [71], whereas *Edis* is required for MB morphology. Of note, *Ect4* knockdown animals did not show any MB morphology defects. This could be due to that either *Ect4* does not play a role in MB formation, or that *Ect4* is involved in MB development but the surviving *Ect4* knockdown flies are “escapers” with a moderate knockdown efficiency. 2) Regarding immune regulation in neurons, *Ect4* and *Edis*, respectively, promotes and suppresses, optimal AMP gene expression (Figs 4B, 4G and 4J, S13A and S16). 3) *Ect4* functions in the tracheal epithelium, but not in the fat body or digestive tract, to dampen the IMD antibacterial signaling (S15 Fig) [64], whereas *Edis* suppresses IMD innate immunity signaling in neurons (Fig 4B and 4G). 4) While *Edis* knockdown consistently leads to hyperactivation of innate immunity signaling both in cultured cells and *in vivo* (Figs 1, 3 and 4), depletion of *Ect4* in cultured S2 cells had no obvious impact (Figs 1J and S7). 5) Even *Ect4* itself displays distinct functions in different tissue/cell types–*Ect4* promotes (Figs 4J and S16) and suppresses (S15 Fig), respectively, optimal AMP gene expression in neurons and tracheal epithelium. Thus elucidating the complex regulatory mechanism underlying *Edis* and *Ect4* expression will facilitate to unravel the function of *Edis* and *Ect4* in diverse cell/tissue types.

Our study suggests that *Edis* encodes a functional protein. We note that our assay involves an artificial circRNA mini-gene. Since the ORF in *Edis* terminates after only 1 nt downstream of the back-spliced exon junction, it does not yield any unique amino acid sequences that could distinguish Edis-p from Ect4, thereby rendering it challenging to employ mass spectrometry to confirm the presence of endogenous Edis-p. In addition, our effort to insert an epitope tag at the endogenous *Edis*-generating exon has been unsuccessful, presumably because such modifications will lead to the addition of exogenous amino acid sequences to the endogenous Ect4 protein, and may cause a growth disadvantage and prevent S2 cells from forming clones. Thus it remains unclear whether endogenous *Edis* can generate Edis-p. Nonetheless, our analysis reveals that ectopic expression of Edis-p not only phenocopies *Edis* overexpression (Fig 6D–6F), but also rescues the innate immunity hyperactivation and neurodevelopmental phenotypes elicited by *Edis* depletion (Fig 7A–7F). In addition, Edis-p co-immunoprecipitates with endogenous Relish and blocks Relish processing both in cells and *in vitro* (Fig 6G–6I). The simplest interpretation is that the immune suppressor function of *Edis*, at least in part, depends on Edis-p (Fig 7G). Thus our study identifies *Edis* as the latest addition to a growing collection of circRNAs that can give rise to functional proteins [59,60].

Proteins encoded by select circRNAs can carry unique amino acid sequences that play a critical role in circRNA function [73]. This is not the case for Edis-p, which is essentially a truncated form of the Ect4 protein (Fig 6A). Edis-p could function as a dominant negative mutant version of Ect4, as Edis-p and Ect4 appear to differentially impact innate immunity gene expression in neurons. These findings could be part of a broader regulatory mechanism by which truncated (and perhaps dominant negative) protein isoforms are generated from products of back-splicing events such as circRNAs. Such mechanism could facilitate to achieve precise fine-tuning of the activity of proteins derived from a given gene locus by taking advantage of the existing transcription and splicing machineries without having to evolve new transcription start sites or novel proteases/protease cleavage sites.

Our study reveals that *Edis* depletion in S2 cells also led to an upregulation of the antifungal peptide gene *Drosomycin* both in the presence and absence of Gram-positive (*M*. *luteus*) bacterial infection (S19A Fig), besides its impact on the antibacterial IMD signaling pathway. Similar phenotypes were observed *in vivo* (S19B Fig). Thus, *Edis* appears to impact both branches of humoral innate immunity in *Drosophila*.

Besides its potent effect on innate immunity, neuron-specific depletion of *Edis* causes impaired locomotor activity and shortened lifespan, accompanied by a severe disruption in brain integrity, consistent with recent findings that aberrant activation of innate immunity induces robust neurodegeneration [35,74]. In *Relish* mutant background, both the elevated *Dpt* gene expression (Fig 4D) and the MB morphology phenotypes of *Elav>shEdis* flies were suppressed. These findings imply that a mild hyperactivation of innate immunity elicited by neuronal *Edis* depletion may be a contributing factor that leads to the age-dependent decline in neural function in *Elav>shEdis* animals. Alternatively, Relish could regulate select neuronal gene(s) that in turn impact neuronal development. It is also possible that hyperactivated innate immunity in *Elav>shEdis* animals might be a consequence of defective neuronal development. For example, immune activation could be elicited by neurotoxic materials or stress signals from *Edis*-depleted neurons. Lastly, hyperactivation of innate immunity and impaired neural development in *Elav>shEdis* flies may be parallel yet independent processes. Further studies will facilitate to distinguish the afore-mentioned alternative non-mutually exclusive models and unravel the mechanism underlying the interplay between innate immunity and neurodevelopment.

In summary, our study identifies the brain-enriched circRNA *Edis* as a negative modulator of innate immunity and an essential regulatory RNA for neural development in *Drosophila*. In particular, our analyses both in cultured cells and *in vivo* demonstrate that *Edis* and its linear sibling *Ect4* are controlled by distinct regulatory mechanisms, which may contribute to the spatiotemporal pattern of their functions. Mechanistically, *Edis* encodes a functional protein with immune suppressor activity. Taken together, our study supports the notion that circRNAs represent a new class of functional regulatory RNAs, provides insights into the molecular mechanism underlying circRNA expression and function, and establishes an animal model that facilitates to unravel the intricate interplay between innate immunity and neurodevelopment.

## Methods

### Statistical analyses

All statistical analyses in this manuscript were performed using biological replicates and the sample number (n) is shown for each dataset in the corresponding legend. Most analyses were performed using student *t*-test, except for survival experiments, which involved the log-rank test, the mushroom body morphology experiments, which involved Chi-squared test, and ChIP analysis, which involved ANOVA test. We note that our sample numbers are not high. In particular, in RT-qPCR assays sample numbers are generally equal to or slightly higher than 3. In our datasets, variations (standard deviation/standard error of the mean) are generally low among biological replicates. Unless noted otherwise, data is shown in this manuscript as mean + standard errors of the mean (SEM), * *p*<0.05; ** *p*<0.01; *** *p*<0.001; **** *p*<0.0001.

### DNA constructs and antibodies

shRNA construct against *Edis*: DNA fragments containing the *shEdis* sequence were amplified by PCR from a DNA oligo and cloned into the Valium 20 vector using NheI and EcoRI restriction sites, to generate Valium 20-sh*Edis*. Overexpression construct for *Edis* in S2 cells: a DNA fragment containing the *Ect4* exon sequence that gives rise to *Edis* was amplified by PCR and cloned into the Hy_pMT Laccase2 MCS exon vector [62], using NheI and SacI restriction sites, to generate Hy_pMT Laccase2-*Edis*. Overexpression construct for *Edis* in flies: the Xho/T4 DNA polymerase/NotI fragment from Hy_pMT Laccase2-*Edis* was ligated to pUAST vector that are sequentially treated with EcoRI/T4 DNA polymerase/NotI, to generate pUAST-*Edis*. A similar strategy was employed to generate the control vector, pUAST-Laccase2 MCS exon vector. Expression vector for Flag-Edis-p: a DNA fragment encoding Flag-tagged Edis-p was amplified by PCR and cloned into pRmHa-3 using EcoRI and BamHI restriction sites, to generate pRmHa-3-Flag-Edis-p. We also generated an shRNA-resistant pRmHa-3-Flag-Edis-p construct in which the 3’ end of the Edis-p ORF was modified at the nucleotide level to encode the same amino acid sequence, for the rescue experiment. A DNA fragment containing the full length Relish ORF was amplified by PCR and cloned into pRmHa-3-Flag using EcoRI and SalI sites to generate the Flag-Relish expression construct. DNA fragments containing Flag- and HA-tagged Ran ORF, respectively, were amplified by PCR and cloned into pRmHa-3 using EcoRI and BamHI sites to generate Flag- and HA-Ran expression constructs. A DNA fragment containing Flag-RpL22 ORF was amplified by PCR and cloned into pRmHa-3 using EcoRI and KpnI sites to generate the Flag-RpL22 expression construct. All constructs were verified by sequencing. Primary antibodies employed in this study include anti-Flag (mouse anti-Flag, F3165, Sigma; rabbit anti-Flag, F7425, Sigma; mouse M2 anti-Flag conjugated agarose beads, A2220, Sigma), rabbit anti-HA (C29F4, Cell Signaling) and anti-Relish antibodies (21F3, Developmental Studies Hybridoma Bank, developed by Dr. Dan Hultmark). Secondary antibodies employed in this study include goat anti-mouse IgG antibody HRP conjugate (12–349, Millipore) and goat anti-rabbit IgG antibody HRP conjugate (12–348, Millipore).

### Dsrna synthesis

Templates for generating dsRNAs were either requested from the DRSC (flyRNAi.org) or amplified by PCR in house. Double-stranded RNAs were synthesized using Megascript *in vitro* transcription kit (Ambion) and purified using RNeasy column (Qiagen).

### Chromatin immunoprecipitation

*Drosophila* S2 cells stably transfected with *sh-w*, *shEdis-A* or *shEdis-B* were transfected with pMT-Flag-Relish plasmid. Cells were treated with copper sulfate for 5 days 24 hours post transfection. The cells were washed with PBS once, then crosslinked with 1% methanol-free formaldehyde for 10 minutes at room temperature. Glycine was added into the cells at a final concentration of 125 mM. Cells were incubated at room temperature for 5 minutes. Cells were subsequently rinsed with 10 mL cold PBS for 3 times, and lysed in 750 μL ChIP lysis buffer (50 mM HEPES, pH7.5, 140 mM NaCl, 1 mM EDTA, pH8.0, 1%Triton X-100, 0.1% Sodium Deoxycholate, 0.1% SDS, protease inhibitors) for 10 minutes. Cells were then sonicated for 15 times for 30 seconds at “High” setting (Bioruptor 300, Diagenode). After sonication cell lysate was centrifugated for 10 minutes at 4°C at a speed of 8000 g. Fifty μL of supernatant aliquot was saved at -20°C as input samples. The remaining supernatants were diluted at 1:10 with RIPA buffer (50 mM Tris-HCl, pH8.0, 150 mM NaCl, 2 mM EDTA, pH8.0, 1% NP-40, 0.5% Sodium Deoxycholate, 0.1% SDS, protease inhibitors). Next 40 μL anti-flag M2 affinity gel (Sigma, A2220) was added into the samples and incubated overnight with rotation at 4°C. Samples were centrifuged for 1 minute at 2000 g and supernatant was removed. Samples were washed sequentially with the following buffers: low salt wash buffer (0.1% SDS, 1% Triton X-100, 2 mM EDTA, 20 mM Tris-HCl, pH8.0, 150 mM NaCl), high salt wash buffer (0.1% SDS, 1% Triton X-100, 2 mM EDTA, 20 mM Tris-HCl, pH8.0, 500 mM NaCl), LiCl wash buffer (0.25 M LiCl, 1 mM EDTA, pH8.0, 1% NP-40, 1% Sodium Deoxycholate, 10 mM Tris-HCl, pH8.0). DNA was eluted with 120 μL elution buffer (1% SDS, 100 mM NaHCO3) by vortexing slowly for 15 minutes at 30°C. Samples were centrifuged for 1 minute at 2000 g and supernatant was transferred into a new tube. Four point eight μL of 5 M NaCl and 2 μL RNase A (10 mg/mL) were added and samples were incubated at 65°C overnight. Two μL proteinase K (20 mg/mL) was added and samples were incubated at 60°C for 1 hour. The DNA was subsequently purified using phenol: chloroform extraction.

### *Drosophila* genetics and infection

Fly stocks are maintained on standard fly food. *UAS-shEdis* transgenic flies were generated using the Valium 20-sh*Edis* construct that integrated into the *attp2* site on the 3^rd^ chromosome (Bestgene). pUAST-*Edis* or the control pUAST-Laccase2 MCS exon vector transgenic flies were generated using the corresponding pUAST constructs (Bestgene). To generate *Edis* knockdown or overexpression flies, we crossed *UAS-shEdis* or pUAST-*Edis* transgenic lines with the *da-Gal4; tub-Gal80*^*ts*^ composite line [43]. Control cross was set up using a shRNA transgenic line, which expresses an artificial *shGFP* RNA, or pUAST-Laccase2 MCS exon vector transgenic flies. To minimize lethality at early developmental stages due to the requirement for *Edis* in development, fly crosses were kept at permissive temperature (18°C) until adult progeny of the appropriate genotype emerge. Subsequently the progeny was shifted to restrictive temperature (29°C) for 5–7 days to allow for transgene expression. We also crossed the shRNA lines to strong ubiquitously expressed drivers *Tubulin-Gal4* and *Actin-Gal4*, or the intermediate ubiquitously expressed driver *da-Gal4*, and kept the crosses at various temperatures (18, 22 and 25°C). These crosses lead to complete and near complete lethality, respectively. To knock down or overexpress *Edis* in different tissues, we crossed the shRNA or pUAST lines to tissue-specific drivers including *Elav*- and *nSyb-Gal4* (neuron), and *Repo-Gal4* (glia). In addition, we employed a similar strategy and used the *UAS-shEct4* and *UAS-dsEct4* lines for depleting the linear *Ect4* in various tissues. For fly infection experiments, male progeny of the appropriate genotype were either left untreated (as control) or pricked with a sharp needle previously dipped in a concentrated suspension of either the Gram-negative bacteria *E*. *coli* or Gram-positive bacteria *M*. *luteus*. Flies were harvested 6 or 24 hours post infection, respectively. Levels of mRNAs encoding the antimicrobial peptide genes such as *Diptericin*, *Drosomcyin*, and the control *RpL32* (*rp49*) mRNA were analyzed by RT-qPCR. To measure host survival and pathogen clearance, infection experiments were carried out by injecting 9.2 nL of bacterial suspension or PBS (control) into flies using Nanoject II (Drummond). For fly survival experiment, a concentrated culture of *Ecc15* (OD_600_ ~5–10) was injected. Survival (in multiple groups of 20–25 flies per group) was monitored daily post infection. To determine pathogen load, a concentrated culture of *Ecc15* (OD_600_ ~5–10) were injected into flies. Subsequently, groups of 4 flies were harvested at various time points post infection and homogenized in 200 μl of sterile PBS. Diluted fly homogenates were plated onto LB plates containing Ampicillin. Colony forming units (CFU) were recorded after 24 hours and CFU per fly is calculated.

### *Drosophila* lifespan and locomotor activity

*UAS-shEdis* or the control *UAS-shGFP* lines were crossed with the *da-Gal4; tub-Gal80*^*ts*^ composite line. Fly crosses were kept at permissive temperature (18°C) until adult progeny of the appropriate genotype emerge. Subsequently, the progeny was shifted to restrictive temperature (29°C) and survival of flies (in multiple groups of 20–25) was monitored daily. For neuron-specific knockdown, *UAS-shEdis* or the control *UAS-shGFP* lines were crossed with the *Elav-Gal4* or *nSyb-Gal4* drivers. Fly crosses were kept at 22°C until adult progeny of the appropriate genotype emerge. Subsequently, the progeny was shifted to 25°C and survival of flies (in multiple groups, 20–25 flies per group) was monitored daily. To measure locomotor activity, we followed the protocol reported by Liu *et al* [75] with minor modifications. Briefly, flies in multiple groups of 15 were placed into conical culture tubes and tapped to the bottom, and the percentage of flies that can climb over the 2-centimeter mark within 15 seconds was recorded.

### Immunofluorescence

For mushroom body staining, adult brains were dissected, stained and imaged as described [76]. Briefly, adult fly heads of proper ages and genotypes were dissected in PTN buffer (0.1 M Sodium Phosphate Buffer, pH 7.2, 0.1% Triton X-100) and fixed with 4% paraformaldehyde (Alfa Aesar, 43368) for 20 minutes at room temperature, followed by two times rinsing in PTN buffer and then three times washing with PTN buffer for 20 minutes each time. Samples were blocked with 5% BSA in PTN buffer at room temperature for 30 minutes and incubated with primary antibody at 4°C for 72 hours, followed by extensive washing as indicated above. Afterwards, samples were incubated with secondary antibody at 4°C overnight, followed by same extensive washing steps showing above. Primary antibodies: mouse anti-Fasciclin II (1D4, Developmental Studies Hybridoma Bank, developed by Dr. Corey Goodman) at 1:50 dilution, rat anti-Elav antibody (7E8A10, Developmental Studies Hybridoma Bank, developed by Dr. Gerald M. Rubin) at 1:100 dilution, prepared in 1XPBT with 5% normal donkey serum to block nonspecific binding. Secondary antibodies: AlexaFluor 488 (Invitrogen, A-21202) or 596-conjugated donkey-anti-rat antibody and AlexaFluor596-conjugated donkey anti-mouse antibody (Invitrogen) at 1:1000 dilution in 1XPBT. Sample were counter-stained with DNA dye 4’,6’-diamidino-2-phenylindole (DAPI) at 1:10,000 dilution of 1mg/mL stock solution in 1XPBT solution for 10 minutes. For phalloidin staining of adult *Drosophila* retinas, the heads were dissected in PBS (137 mM NaCl, 2.7 mM KCl, 10 mM Na2HPO4, 2 mM KH2PO4, pH 7.4) and fixed with 4% paraformaldehyde for 1 hour. Subsequently, retinas were dissected and fixed for an additional 30 minutes. Retinas were rinsed three times with PBST (PBS + 0.4% Triton X-100) and incubated with 100 nM phalloidin (Cytoskeleton Inc., 057) overnight at 4°C, followed by three times washing. For giant fiber system of adult brain, the heads were dissected in PBS and fixed with 4% paraformaldehyde for 20 minutes, followed by three times washing with PBS. Then all the prepared samples were mounted in 80% glycerol and imaged using a Zeiss compound fluorescent microscope (Zeiss Axio Imager Z1) and processed by Zen software (Zeiss), or Nikon confocal microscope (Nikon A1, Tokyo, Japan). To visualize synapses, immunocytochemistry of dissected 3^rd^ instar larvae was performed as described previously [77]. The rabbit anti-DVGlut (*Drosophila* vesicular glutamate transporter) was described previously [78] and used at 1:5,000 dilution. Goat anti-HRP antibody was obtained from Jackson Immunoresearch and used at 1:1000 dilution (123-165-021).

For immunostaining of cultured cells, S2 cells stably transfected with pMT-Flag-Edis-p or pMT vector were transfected with pMT-Relish-HA. Two days post transfection cells were treated with 200 μM CuSO_4_ for 2 days. Cells were washed by PBS and fixed in 4% formaldehyde for 10 min at room temperature. After washing with PBST, cells were blocked with 2% BSA in PBST for 30 min. Cells were subsequently incubated with anti-Flag antibody (1:1000, Sigma, F3165) and anti-HA (1,1000, Cell Signaling Technology, #C29F4) overnight at 4°C, washed and incubated in secondary antibodies (anti-mouse 488, 1:1000; anti-Rabbit 594, 1:1000) for 1 hour in room temperature and washed with PBST. Images were acquired on a Nikon confocal microscope. Fluorescence intensity was analyzed by NIS-Elements Viewer.

### Natural infection of larvae and tracheal branch imaging

We followed the protocol of Akhouayri *et al* with minor modifications [64]. An overnight *Ecc15* culture was subjected to centrifugation at 4,000 g for 10 minutes at 4°C. The pellet was resuspended in fresh LB liquid media. Around 200 μL of the resulting cell paste (estimated OD_600_  =  200) was directly added on top of the standard medium containing feeding third instar larvae at 25°C. A similar volume of LB broth was used as a control. After 24 hours, the fed larvae were placed in a drop 100% glycerol on glass microscope slide. The slide was heated at 70°C for 10 seconds, and *Drosomycin* transcription was monitored by fluorescence analysis using *Drs-GFP* reporter.

### Cell culture, transfection, and RNAi

*Drosophila* S2 cells are maintained in Schneider’s medium (Invitrogen) supplemented with 10% fetal bovine serum (FBS) and 1% penicillin-streptomycin (Invitrogen). S2 cells stably expressing *shEdis* were generated by transfection with Valium 20-sh*Edis*, pMT-Gal4, and the selection marker plasmid pHS-neo using the calcium phosphate method, followed by selection in medium containing 400 μg/mL G418 (Calbiochem). A control cell line expressing shRNA targeting the *w* gene serves as control. To overexpress *Edis*, S2 cells were transfected with Hy_pMT Laccase2-*Edis*, followed by selection in medium containing 400 μg/mL hygromycin (Calbiochem). Cells stably transfected with Hy_pMT Laccase2 MCS exon vector serve as control. Cells were grown in culture medium containing CuSO_4_ (final concentration 250 μM) for ~3 and 5 days, respectively, to achieve *Edis* overexpression and knockdown. For transient knockdown in S2 cells, dsRNA treatment was performed as described previously [36–38]. Briefly, ~2 × 10^6^ S2 cells were seeded in 6-well plates for 24 hours and then transfected with 3 μg of the appropriate dsRNA using the calcium phosphate protocol. Two days later, the cells were harvested, replated in 6-cm plates for 24 hours, and then transfected again with another 9 μg of dsRNA. Cells were harvested three days after the second dsRNA treatment.

### Peptidoglycan, bacterial and ecdysone treatment in S2 cells

To generate samples for RT-qPCR analysis, *Drosophila* S2 cells were treated with 1 μM 20-hydroxyecdysone (Sigma) for 24 hours. Subsequently, cells were treated with 10 μg/mL crude LPS prep (Sigma), which contains peptidoglycan (PGN) as a potent inducer of IMD signaling [17], for another 3 to 6 hours. To generate samples for RNA-seq analysis, S2 cells (at ~ 2X10^6^ cells/mL) were 1) left untreated, 2) treated with 1 μM 20-hydroxyecdysone (Sigma) for 24 hours, 3) treated with a mixture of overnight cultures of *E*. *coli 1106* and *M*. *luteus* for 48 hours, or 4) treated with 1 μM 20-hydroxyecdysone for 24 hours and subsequently with a mixture of overnight cultures of *E*. *coli 1106* and *M*. *luteus* for an additional 48 hours. Total RNA was extracted and levels of the antimicrobial peptide genes *Diptericin*, *Attacin A*, *Metchnikowin* and the control *RpL32* mRNA were analyzed by RT-qPCR. For Relish processing experiments, cells were first treated with 20-hydroxyecdysone (Sigma) for 24 hours. Subsequently, cells were treated with 10 μg/mL crude LPS prep for 10–15 minutes. Cell lysates were prepared and subjected to immunoblot using anti-Relish antibody. The ratio of cleaved/full length Relish was employed for quantification of Relish processing. For *Edis* overexpression and knockdown experiments, the corresponding stable cells were first treated with CuSO_4_ (250 μM) for ~3 and 5 days, respectively, to achieve *Edis* overexpression and knockdown, prior to 20-hydroxyecdysone and PGN treatment.

### *In vitro* Relish processing

Purification of recombinant proteins was processed as previously described [72]. Briefly, 50 mL of stably transfected cells (Flag-Dredd, Flag-Ran or Flag-Edis) were plated at a density of 10^6^ cells/mL. After 24 hours, cells were treated with 500 μM copper sulfate for 24 hours and cells were collected and lysed in 1.5 mL lysis buffer. The supernatants were incubated with 100 μL anti-Flag-agarose beads (Sigma) overnight and then the beads were eluted with 300 μL 3XFlag-Peptide (100 μg/mL; sigma). The eluates were passed through a 10-kDa cut-off filter (Millipore). For *in vitro* caspase assays, S2 cells were lysed in 100 μL lysis buffer per 5 mL of saturated cell culture. Twenty microliters of cell lysate was incubated with various combinations of 10 uL purified Flag-Edis (70 ng), Flag-Ran (80 ng), 10 uL Flag-Dredd (30 ng) and 20 μL 3Xcaspase buffer in a 60 μL reaction system at 30 °C for 2h. The reactions were subsequently subjected to immunoblot analysis using anti-Relish and anti-Flag antibodies.

### Protein immunoprecipitation and immunoblotting

Cells were lysed in lysis buffer (20 mM Tris-HCl (pH 7.6), 150 mM NaCl, 2 mM EDTA, 10% glycerol, 1% Triton X-100, 1 mM DTT, 1 mM orthovanadate) supplemented with protease inhibitor cocktail (Roche). Cleared total lysates were immunoprecipitated with antibodies against Flag (Sigma F3165). Both input and immunoprecipitated samples were analyzed by SDS-PAGE followed by immunoblotting with antibodies against Flag (Sigma, F7425, 1:5000) and Relish (DSHB, 21F3, 1:1000).

### Reporter assay

Briefly, ~5×10^5^ of afore-mentioned S2 stable cells were seeded in 24-well plates the day before transfection. Subsequently, 500 ng *att-luc* reporter construct (firefly luciferase reporter gene driven by the promoter of the antimicrobial peptide gene *Attacin*) and 20 ng of *actin-Renilla luciferase* were transfected into these cells. Two days post transfection, cells were treated with 250 μM copper for ~3 and 5 days, respectively, to achieve *Edis* overexpression and knockdown. Next, cells were treated with 1 μM 20-hydroxyecdysone (Sigma) for 24 hours and subsequently left untreated or treated with PGN for 6 hours. Cell suspensions were arrayed in 384-well plates and reporter activity was measured using the Dual-Glo system (Promega). For data processing, firefly/*Renilla* ratio was calculated and normalized against the control sample.

### RNase R treatment and RT-qPCR

Two protocols were used. 1) We followed the protocol of Salzman *et al*. Total RNA was isolated with TRIzol, either treated with RNase R at 37°C for 1 hour or mock treated. The RNA samples were subsequently reverse transcribed using Superscript III (Invitrogen) and random hexamer primers, and levels of circular and linear RNAs were measured by quantitative PCR. Levels of circular and linear RNAs prior to and after RNase R treatment were compared. 2) We followed the protocol of Rybak-Wolf *et al* with minor modifications: Total RNA was treated with 3U/μg RNase R (RNR07250, Lucigen) for 15 minutes at 37°C or mock treated. The RNA was immediately transferred to ice, spiked with 10% total RNA from mouse brain. Subsequently, the RNA samples were subjected to acidic phenol extraction, ethanol precipitation, and reverse transcription. Levels of circular and linear RNAs were quantified by quantitative PCR. Levels of mouse *gapdh* transcript were employed for normalization. Quantitative PCR was performed using the iQ SYBR-green reagents on a CFX96 Real-Time PCR Detection System (Bio-Rad). Fold changes in RNA levels were calculated using the ΔΔCt method.

### Circular RNA identification and RNA-seq Data Analysis

Raw reads (fastq files) were pre-processed by trimming the adapter sequences and filtering out the low-quality reads (reads with base quality < = 20). The detection of circular RNA was based on the computational pipeline described in Memczak *et al* [46]. The raw reads (paired-end, 2x100) were mapped to *Drosophila* reference genome (dm3) using bowtie2 [79]. The *find_circ* program with the same parameters recommended by developer was applied for detecting circRNAs using those unmapped reads [46].

#### Oligonucleotides

See S6 Table.

#### *Drosophila* stocks

See S7 Table.

## Supporting information

S1 Fig Mapping of circular RNAs to annotated exons and introns. The majority of identified circular RNA candidates (58.5%) from S2 cells that have been treated with both 20-hydroxyecdysone and a mixture of *Escherichia coli* and *Micrococcus luteus* are composed of single exons, whereas a significant proportion (37.8%) contains two or more exons. A small fraction of circular RNA candidates (2.1%) map exclusively to annotated introns. However, they are predominantly derived from the anti-sense strand to the annotated transcript and likely a result of cryptic splicing events, since consensus splice junction sequences (5’-GU and 3’-AG) were found to flank the region that gives rise to these circular RNAs. The remaining circular RNA candidates (1.6%) were collectively sorted into the “miscellaneous” category.
(TIF)

S2 Fig Identification and validation of circRNAs. **(A)** A schematic of circRNA formation. **(B)** The circRNA *circ_43838* and its host gene *CG17646* are shown. Splice donor and acceptor sites (*GT* and *AG*) are in red and orange, respectively, whereas exons 2 and 3 are in blue and green, respectively. Sanger sequencing confirms head-to-tail splicing. Convergent and divergent primers are shown on the top. **(C-D)** Divergent primers amplify circRNAs in cDNA (**c**) but not genomic DNA (**g**), whereas convergent primers amplify both linear and circular RNAs from both templates. Φ, no template control; M, markers. **(E-F)** circRNAs are resistant to RNase R treatment. Levels of indicated circular RNAs and their linear siblings were quantified by qPCR prior to and after RNase R treatment. Two protocols were employed: in **E**, levels of the indicated RNAs were quantified directly based on qPCR results. In **F**, After RNase R treatment and prior to reverse transcription, a small amount of mouse brain total RNA was added as “spike-in” controls. Levels of the indicated RNAs were normalized to that of the mouse *gapdh* mRNA (student t test, n = 3; all data herein are presented as mean + standard error of the mean (**SEM**), * *p*<0.05; ** *p*<0.01; *** *p*<0.001; ns, non-significant). The apparent enrichment of circRNA in +RNase R samples in **E** is likely a result of higher reverse transcription efficiency due to linear RNA depletion.
(TIF)

S3 Fig Select circRNAs impact innate immunity in cultured S2 cells. S2 cells were transfected with shRNA constructs targeting the back-spliced exon junction of individual circRNAs, a positive control shRNA targeting *IMD*, or a negative control (Ctrl) shRNA against the *white* gene, treated with 20-hydroxyecdysone (**20-HE**) and PGN. Levels of *Diptericin* (***Dpt***) mRNA were measured by RT-qPCR and normalized to *RpL32* (student t test, n = 3, * *p*<0.05; ** *p*<0.01; *** *p*<0.001).
(TIF)

S4 Fig *Edis* is derived from the *Ect4* locus. A schematic of the *Ect4* locus that gives rise to *Edis*. The locations of the divergent/convergent primer pairs and the *Edis*-generating exon are shown.
(TIF)

S5 Fig *Edis* levels are not affected by ecdysone treatment or bacterial infection. S2 cells were either left untreated or treated with various combinations of 1 μM 20-hydroxyecdysone and/or a mixture of overnight cultures of *E*. *coli 1106* and *M*. *luteus*. Total RNA was extracted and levels of *Edis* (**A**), the antimicrobial peptide genes *Diptericin* (**B**), and the control *RpL32* mRNA were analyzed by RT-qPCR (student t test, n = 3, * *p*<0.05; ns, non-significant).
(TIF)

S6 Fig *Edis* depletion impacts innate immunity signaling. (**A**) S2 cells were transfected with two independent shRNA constructs **(***sh-Edis-A* or -*B*) targeting the back-spliced exon junction of *Edis* or a control (Ctrl) shRNA against the *white* gene, treated with 20-hydroxyecdysone (**20-HE**) and subsequently with (+) or without PGN (-). Levels of *Edis* and *Diptericin* (***Dpt***) mRNA were measured by RT-qPCR and normalized to *RpL32* (student t test, n = 3, * *p*<0.05; ** *p*<0.01). (**B-D**) To examine potential off target effects of the shRNAs against *Edis*, expression of candidate off target effect genes (see S5 Table) were measured by RT-qPCR and normalized to *RpL32* (student t test, n = 3, * *p*<0.05).
(TIF)

S7 Fig *Ect4* depletion does not affect IMD innate immunity signaling in S2 cells. S2 cells were transfected with two independent dsRNAs targeting exclusively the linear *Ect4* transcript, a positive control dsRNA against *ird5* or a negative control dsRNA targeting *LacZ*. Cells were treated with 20-HE and levels of linear *Ect4*, *Dpt* and *Drs* mRNA were measured and normalized against *RpL32* (student t test, n = 3, * *p*<0.05; ** *p*<0.01; *** *p*<0.001).
(TIF)

S8 Fig *Edis* overexpression blocks IMD innate immunity signaling. S2 cells were transfected with constructs expressing *Edis* **(A-B)** or *circ_714* (**C**-**D**). Cells were treated with 20-HE and PGN. Levels of the *Edis* or *circ_714* circular RNAs and the *Dpt* mRNA were measured and normalized to *RpL32* (student t test, n = 3, * *p*<0.05; ** *p*<0.01). Cells transfected with an empty vector served as control. (**E**) Blast search against the *Drosophila melanogaster* genome using the *Edis* primer sequence pulled out a number of loci besides *Ect4*. To demonstrate specificity of the *Edis* primers, we chose the following criteria in selecting loci for further analysis: 1) 14 nt or more overlap; 2) the overlaps map to exons of annotated transcripts; and 3) the overlapping region is located within 3 nts of the 3’ end of the *Edis* primer. *Edis* overexpression does not affect expression of these genes (student t test, n = 3, * *p*<0.05; *** *p*<0.001).
(TIF)

S9 Fig *Edis* regulates innate immunity *in vivo*. (**A-B**) Flies carrying the ubiquitously expressed *daughterless-Gal4* driver and a temperature-sensitive *Gal80* were crossed to *UAS-shEdis* or control *shGFP* flies. Fly crosses were kept at 18°C. Adult progeny were collected and either kept at 18°C or shifted to 29°C for 5 days to allow for shRNA transgene expression. Total RNAs were extracted and levels of the circular RNA *Edis* (**A**) and *Dpt* (**B**) were measured (student t test, n≥3, * *p*<0.05; *** *p*<0.001; ns, non-significant).
(TIF)

S10 Fig Ubiquitous *Ect4* depletion does not affect global expression of antimicrobial peptide genes. Flies carrying the ubiquitously expressed *daughterless-Gal4* driver and a temperature-sensitive *Gal80* were crossed to *UAS-Dcr2; UAS-dsEct4* or control *UAS-Dcr2; shGFP* flies. Fly crosses were kept at 18°C. Adult progeny were shifted to 29°C for 5 days to allow for transgene expression. Total RNAs were extracted and levels of linear *Ect4*, circular *Edis* and *Dpt* transcripts were measured (student t test, n≥3, * *p*<0.05; ns, non-significant).
(TIF)

S11 Fig Ubiquitous or neuron-specific *Edis* depletion leads to hyperactivation of innate immunity, impaired locomotion and shortened lifespan. (**A**) female *da>shEdis; Gal80*^*ts*^ flies and control *da>shGFP; Gal80*^*ts*^ flies were kept at 29°C for indicated period of time (below). Flies in groups of 15 were placed into conical culture tubes and tapped to the bottom, and the percentage of flies that can climb over the 2-centimeter mark within 15 seconds was recorded and shown (student t test, n≥4, * *p*<0.05; *** *p*<0.001). (B) Female *da>shEdis; Gal80*^*ts*^ flies and control *da>shGFP; Gal80*^*ts*^ flies in multiple groups of 25 were kept at 29°C. Fly survival was recorded daily and plotted (log rank test, n≥6, **** *p*<0.0001). (C-D) The *UAS-shEdis* or control *UAS-shGFP* flies were crossed to the neuron-specific *Elav-Gal4* driver line. Locomotor activity (C) and lifespan (D) of 1 to 4 week old female *Elav>shEdis* and control flies were measured and shown (student t test in C and log rank test in D, n = 4 to 8, *** *p*<0.001; **** *p*<0.0001).
(TIF)

S12 Fig Impact of *Edis* depletion on antibacterial innate immunity in various tissues. (**A**) Total RNA was extracted from either whole flies or various dissected tissues, and levels of *Edis* and *Ect4* were measured by RT-qPCR and normalized to the control *RpL32* mRNA (student t test, n = 3). (**B**) The *UAS-shEdis* or control *UAS-shGFP* flies were crossed to the glia-specific *Repo-Gal4* driver line. Total RNA was prepared from dissected fly heads, and levels of the *Dpt* mRNA and *Edis* were measured (student t test, *** *p*<0.001; ns, non-significant).
(TIF)

S13 Fig Neuron-specific *Edis* depletion using a second shRNA transgene leads to innate immunity hyperactivation and MB morphology phenotypes. (**A**) Control *UAS-shgfp* or *UAS-shEdis-B* flies were crossed to the neuron-specific *Elav-Gal4* driver animals. Total RNA samples were prepared from fly heads of the indicated genotypes. Levels of the *Edis*, *Ect4* and *Dpt* transcripts were measured and normalized to *rp49* (student t test, n≥3, *** *p*<0.001; ns, non-significant). (**B**) Shown are confocal images of dorsal anterior regions of adult brains with neuron-specific expression of control *shgfp* (left panel) or *shEdis-B* (middle and right panels) driven by the neuron-specific *Elav-Gal4* driver. In control brain (left panel), anti-FasII antibody delineates the central complex as well as the vertical α and horizontal β and γ lobes of the mushroom bodies (**MB**s), with γ lobes showing weaker FasII signal, as indicated. Depletion of *Edis* resulted in a spectrum of severe morphological defects in the MBs, including missing (red circles) and/or fused lobes (red arrows). Scale bar: 20 μm. **(C)** Quantification of MB morphology phenotypes shown in **B** (Chi-squared test, sample numbers are shown on top, *** *p*<0.001). Percentage of animals displaying normal MB morphology is shown.
(TIF)

S14 Fig Neuron-specific *Edis* depletion leads to hyperactivation of innate immunity. The *UAS-shEdis* or control *UAS-shGFP* flies were crossed to the neuron-specific *Elav-Gal4* driver line. Levels of the circRNA *Edis* or the *Dpt* mRNA in male or female fly head samples were measured (student t test, n = 3, * *p*<0.05; ** *p*<0.01; *** *p*<0.001).
(TIF)

S15 Fig Depletion of *Ect4* leads to elevated antimicrobial peptide gene expression in tracheal epithelium. The control *UAS-shmCherry* or *UAS-dsEct4* flies were crossed to the *UAS-Dcr-2 breathless (btl)-Gal4* driver line. A *Drosomycin* (*Drs*) promoter driven *GFP* transgene was also present in the genetic background, to visualize the activation of antimicrobial peptide genes. Progeny larvae were fed with food containing *Ecc15* for 24 hours. Levels of GFP signal in the trachea of third instar larvae were examined (n = 3). Representative images are shown. VB: visceral branch, DT: dorsal trunk, FB: fat body.
(TIF)

S16 Fig Neuron-specific depletion of *Edis*, but not its linear sibling *Ect4*, leads to hyperactivation of innate immunity. The *UAS-shEdis* (**A**-**B**) or *UAS-dsEct4* (**C**-**D**) flies were crossed to the *UAS-Dcr-2 Elav-Gal4* driver line. Total RNAs were extracted from male fly head samples and reverse transcribed using either random primers (**A**,**C**) or oligo dT (**B**,**D**). Levels of the circRNA *Edis*, linear *Ect4* or the *Dpt* mRNA were measured (student t test, n = 3, * *p*<0.05; ** *p*<0.01; *** *p*<0.001; ns, non-significant). Samples from a cross between *UAS-shGFP* and *UAS-Dcr-2 Elav-Gal4* driver line serve as controls. As expected, we detected consistent changes in levels of the linear *Ect4* and *Dpt* mRNAs regardless of the type of oligos employed in the reverse transcription systems, whereas changes in levels of the circRNA *Edis* that were previously detected in random primer-based reverse transcription reactions were no longer obvious in oligo d(T)-based reverse transcription reactions, as the circRNA *Edis* was not expected to be efficiently reverse transcribed using oligo d(T). (**E**) The *UAS-shGFP* or *UAS-shEct4* flies were crossed to the *Elav-Gal4* driver line. Total RNAs were extracted from male fly head samples and reverse transcribed using either random primers. Levels of the circRNA *Edis*, linear *Ect4* or the *Dpt* mRNA were measured (student t test, n = 3, *** *p*<0.001; ns, non-significant).
(TIF)

S17 Fig *Edis* depletion does not impact the expression or stability of Flag-Ect4. (**A**) S2 cells stably transfected with the *shEdis* or control shRNA constructs were transfected with Flag-Ect4 and HA-Ran expression constructs. Cells were treated with copper to induce transgene expression, and subjected to immunoblot assay using anti-Flag (upper panel) and anti-HA (lower panel) antibodies. (**B**) The ratio of Flag-Ect4/HA-Ran in **A** was quantified and shown (student t test, n = 3, ns, non-significant).
(TIF)

S18 Fig Epitope-tagged Edis-p and Relish co-localize in S2 cells. S2 cells were transfected with various combinations of pMT, pMT-Flag-Edis-p and pMT-Relish-HA constructs, as indicated on the left. Epitope-tagged proteins were visualized by staining with antibodies against Flag (green) and HA (red) epitopes. Nuclei were visualized by DAPI staining (blue). Columns from left to right show DAPI, Flag, HA signals and overlay of the three channels, respectively. Arrows point to co-localization between Flag-Edis-p and Relish-HA. Shown on the right are quantifications of signals of various channels along a line that crosses the region pointed by the arrows.
(TIF)

S19 Fig Depletion of *Edis* impacts anti-fungal Toll signaling. (**A**) S2 cells were transfected with two independent shRNA constructs **(***sh-Edis-A* or -*B*) targeting the back-spliced exon junction of *Edis* or a control (Ctrl) shRNA against the *white* gene. Cells were treated with 20-hydroxyecdysone (**20-HE**) and subsequently with (+) or without (-) the Gram-positive bacterial *M*. *luteus*. Levels of *Diptericin* (***Dpt***), *Drosomycin (****Drs****)* mRNAs and *Edis* were measured by RT-qPCR and normalized to *RpL32* (student t test, n = 3, * *p*<0.05; ** *p*<0.01; *** *p*<0.001). (**B**) Flies carrying the ubiquitously expressed *daughterless-Gal4* driver and a temperature-sensitive *Gal80* were crossed to *UAS-shEdis* or control *shGFP* flies. Fly crosses were kept at 18°C. Adult progeny were collected and shifted to 29°C for 5 days to allow for shRNA transgene expression. Flies were either left un-infected or pricked with a needle previously dipped into a concentrated culture of the Gram-positive bacteria *M*. *luteus*. Flies were collected 24 hours later and levels of the circular RNA *Edis* and *Drosomycin* (*Drs*) were measured (student t test, n = 3; n = 1 for non-infected da>shgfp sample, *** *p*<0.001).
(TIF)

S1 Table Candidate circRNAs identified from S2 cells. Shown are the genome coordinates, host genes, names, number of read counts, strand polarity and number of unique read counts for circRNA candidates identified from S2 cells treated with various combinations of 20-HE and a mixture of *Escherichia coli* and *Micrococcus luteus*. C1-S2: naïve S2 cells; C2_20E: S2 cells treated with 20-HE only; C3_EML: S2 cells treated with a mixture of *Escherichia coli* and *Micrococcus luteus* only; C4_20EML: S2 cells treated with both 20-HE and a mixture of *Escherichia coli* and *Micrococcus luteus*.
(XLSX)

S2 Table Comparison of circular RNA candidates with previous published datasets. (XLSX)

S3 Table The intron and exon composition of circRNA candidates identified in S2 cells treated with both 20-hydroxyecdysone and a mixture of *Escherichia coli* and *Micrococcus luteus*. The majority of circular RNA candidates (58.5%) are composed of single exons, whereas a significant proportion (37.8%) contains two or more exons. A small fraction of circular RNA candidates (2.1%) map exclusively to annotated introns. The remaining (1.6%) were collectively sorted into the “miscellaneous” category.
(XLSX)

S4 Table Validated circRNAs from S2 cells. Shown are the genome coordinates, host genes, names, number of read counts, strand polarity and number of unique read counts for validated circRNAs.
(XLSX)

S5 Table Off target analysis of sh-*Edis*. (XLSX)

S6 Table Sequences of oligonucleotides employed in this study. (XLSX)

S7 Table *Drosophila* stocks employed in this study. (XLSX)

S1 Data Data underlying the figures. (XLSX)

S2 Data Microscopic, PCR and immunoblot images. (RAR)
