# Supplementary figures and images for: The circular RNA Edis regulates neurodevelopment and innate immunity
Source: PLoS Genet. 2022 Oct 27;18(10):e1010429. doi: 10.1371/journal.pgen.1010429 (PMC9612488; doi:10.1371/journal.pgen.1010429)

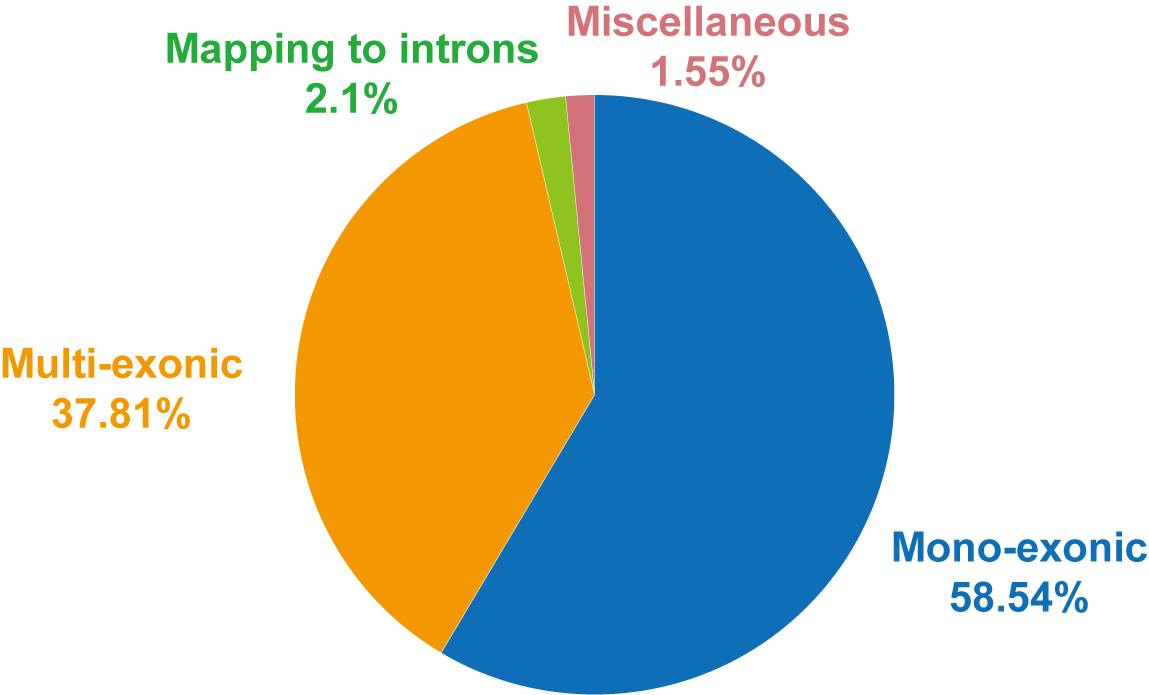

Supplement: S1 Fig — The majority of identified circular RNA candidates (58.5%) from S2 cells that have been treated with both 20-hydroxyecdysone and a mixture of Escherichia coli and Micrococcus luteus are composed of single exons, whereas a significant proportion (37.8%) contains two or more exons. A small fraction of circular RNA candidates (2.1%) map exclusively to annotated introns. However, they are predominantly derived from the anti-sense strand to the annotated transcript and likely a result of cryptic splicing events, since consensus splice junction sequences (5’-GU and 3’-AG) were found to flank the region that gives rise to these circular RNAs. The remaining circular RNA candidates (1.6%) were collectively sorted into the “miscellaneous” category. (TIF) [file pgen.1010429.s001.tif]

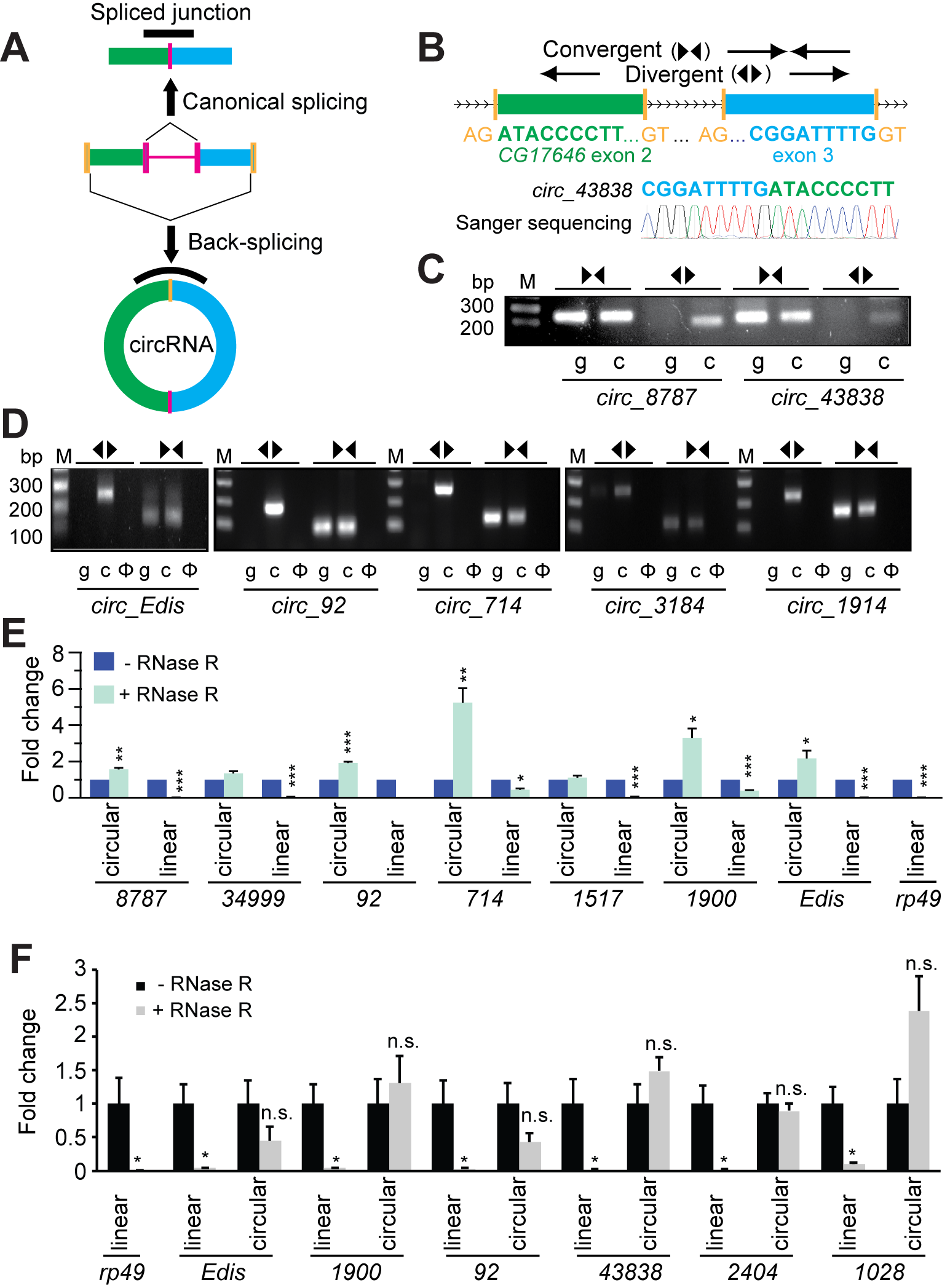

Supplement: S2 Fig — (A) A schematic of circRNA formation. (B) The circRNA circ_43838 and its host gene CG17646 are shown. Splice donor and acceptor sites (GT and AG) are in red and orange, respectively, whereas exons 2 and 3 are in blue and green, respectively. Sanger sequencing confirms head-to-tail splicing. Convergent and divergent primers are shown on the top. (C-D) Divergent primers amplify circRNAs in cDNA (c) but not genomic DNA (g), whereas convergent primers amplify both linear and circular RNAs from both templates. Φ, no template control; M, markers. (E-F) circRNAs are resistant to RNase R treatment. Levels of indicated circular RNAs and their linear siblings were quantified by qPCR prior to and after RNase R treatment. Two protocols were employed: in E, levels of the indicated RNAs were quantified directly based on qPCR results. In F, After RNase R treatment and prior to reverse transcription, a small amount of mouse brain total RNA was added as “spike-in” controls. Levels of the indicated RNAs were normalized to that of the mouse gapdh mRNA (student t test, n = 3; all data herein are presented as mean + standard error of the mean (SEM), * p<0.05; ** p<0.01; *** p<0.001; ns, non-significant). The apparent enrichment of circRNA in +RNase R samples in E is likely a result of higher reverse transcription efficiency due to linear RNA depletion. (TIF) [file pgen.1010429.s002.tif]

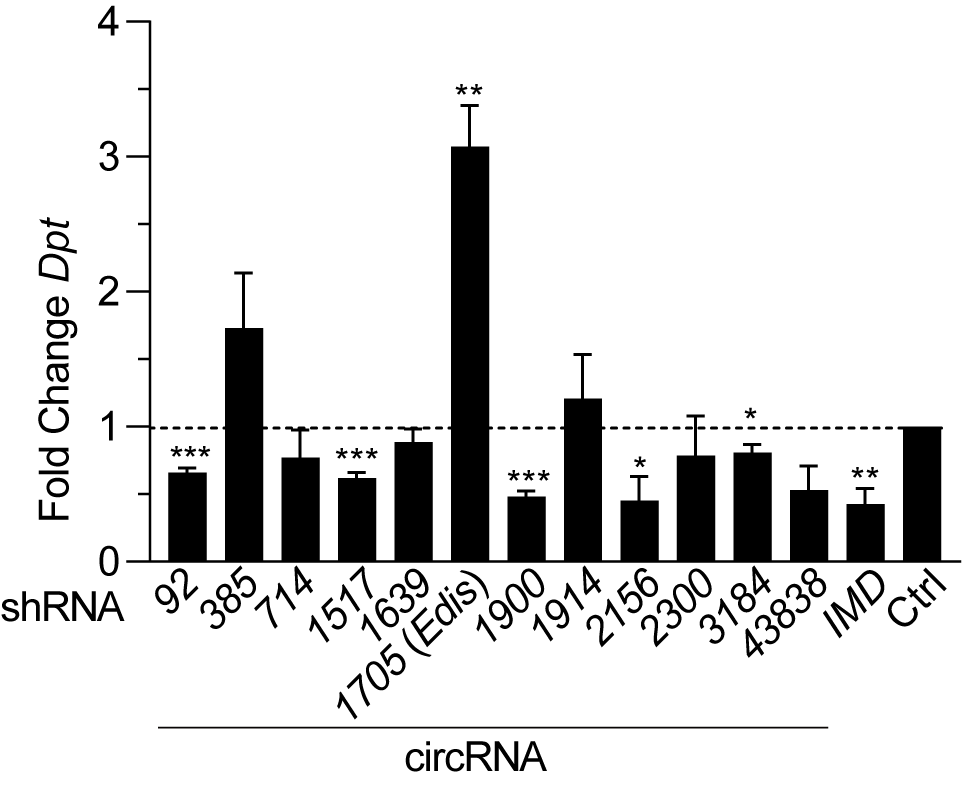

Supplement: S3 Fig — S2 cells were transfected with shRNA constructs targeting the back-spliced exon junction of individual circRNAs, a positive control shRNA targeting IMD, or a negative control (Ctrl) shRNA against the white gene, treated with 20-hydroxyecdysone (20-HE) and PGN. Levels of Diptericin (Dpt) mRNA were measured by RT-qPCR and normalized to RpL32 (student t test, n = 3, * p<0.05; ** p<0.01; *** p<0.001). (TIF) [file pgen.1010429.s003.tif]

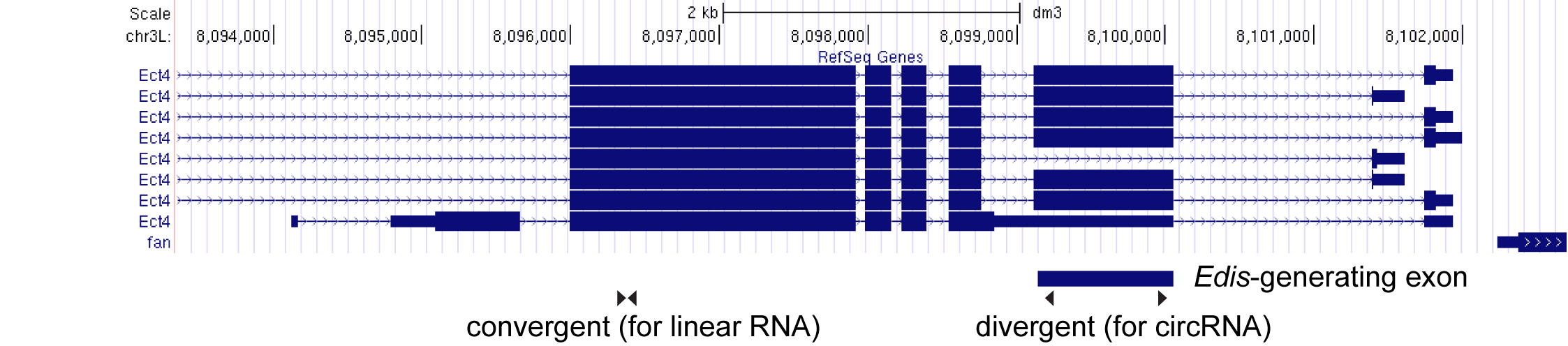

Supplement: S4 Fig — A schematic of the Ect4 locus that gives rise to Edis. The locations of the divergent/convergent primer pairs and the Edis-generating exon are shown. (TIF) [file pgen.1010429.s004.tif]

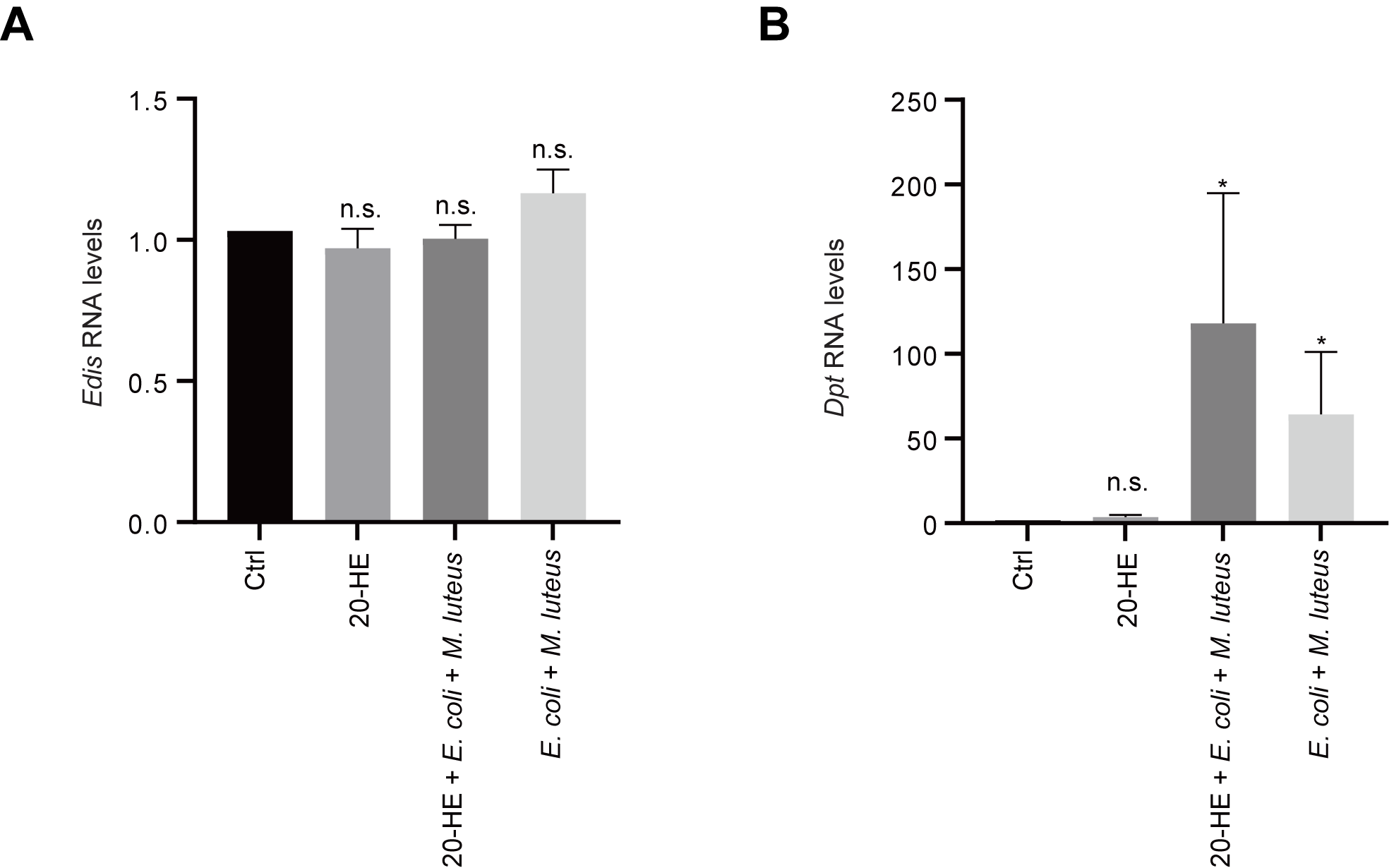

Supplement: S5 Fig — S2 cells were either left untreated or treated with various combinations of 1 μM 20-hydroxyecdysone and/or a mixture of overnight cultures of E. coli 1106 and M. luteus. Total RNA was extracted and levels of Edis (A), the antimicrobial peptide genes Diptericin (B), and the control RpL32 mRNA were analyzed by RT-qPCR (student t test, n = 3, * p<0.05; ns, non-significant). (TIF) [file pgen.1010429.s005.tif]

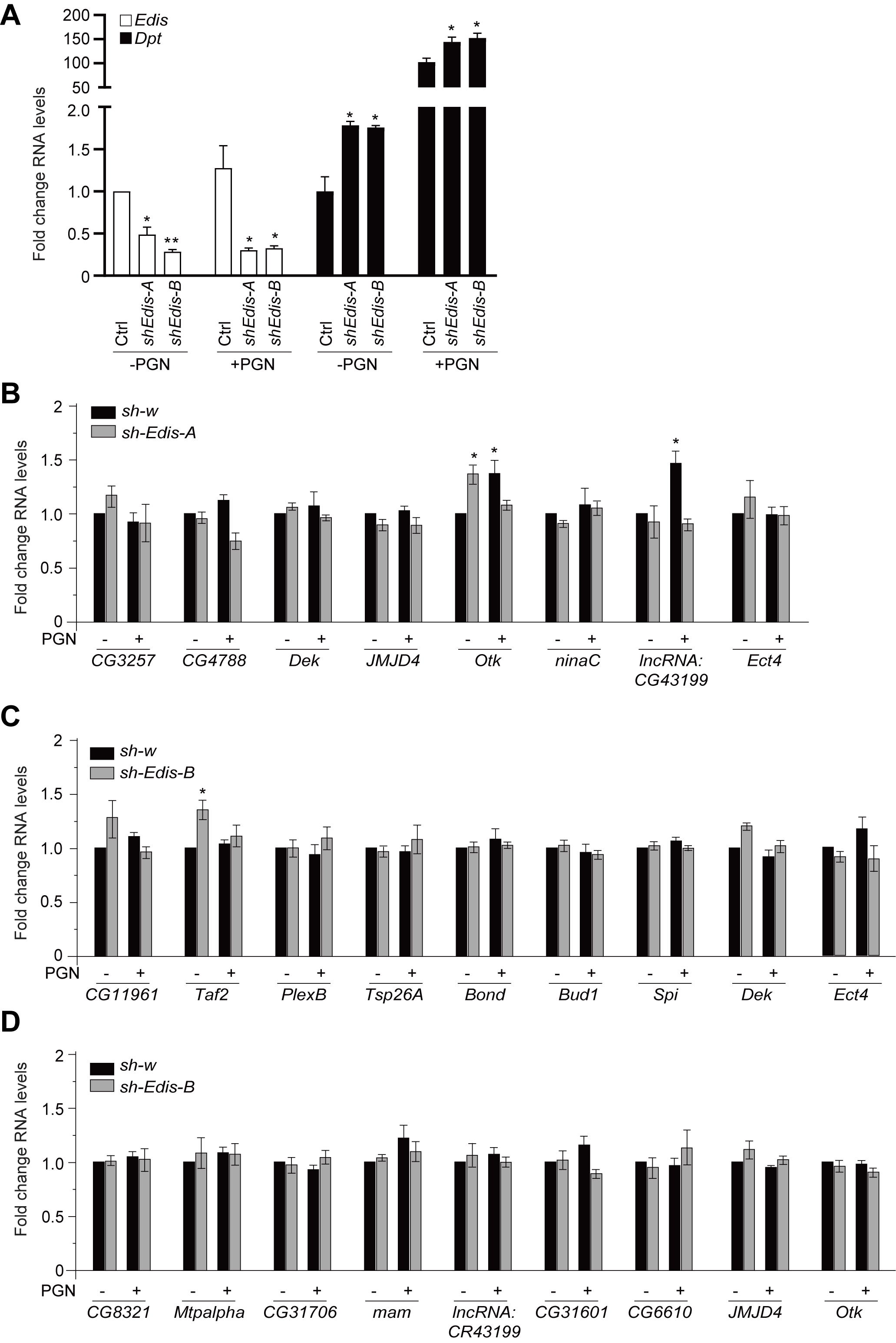

Supplement: S6 Fig — (A) S2 cells were transfected with two independent shRNA constructs (sh-Edis-A or -B) targeting the back-spliced exon junction of Edis or a control (Ctrl) shRNA against the white gene, treated with 20-hydroxyecdysone (20-HE) and subsequently with (+) or without PGN (-). Levels of Edis and Diptericin (Dpt) mRNA were measured by RT-qPCR and normalized to RpL32 (student t test, n = 3, * p<0.05; ** p<0.01). (B-D) To examine potential off target effects of the shRNAs against Edis, expression of candidate off target effect genes (see S5 Table) were measured by RT-qPCR and normalized to RpL32 (student t test, n = 3, * p<0.05). (TIF) [file pgen.1010429.s006.tif]

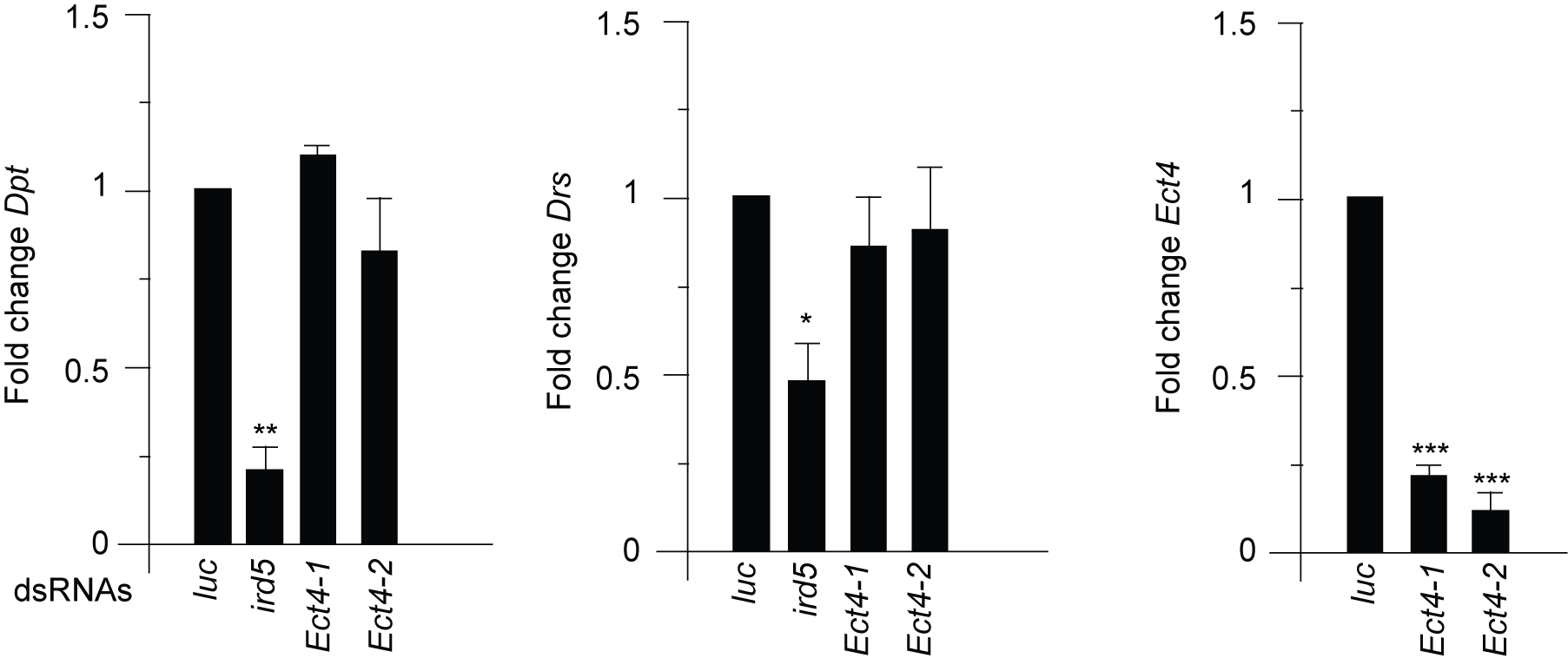

Supplement: S7 Fig — S2 cells were transfected with two independent dsRNAs targeting exclusively the linear Ect4 transcript, a positive control dsRNA against ird5 or a negative control dsRNA targeting LacZ. Cells were treated with 20-HE and levels of linear Ect4, Dpt and Drs mRNA were measured and normalized against RpL32 (student t test, n = 3, * p<0.05; ** p<0.01; *** p<0.001). (TIF) [file pgen.1010429.s007.tif]

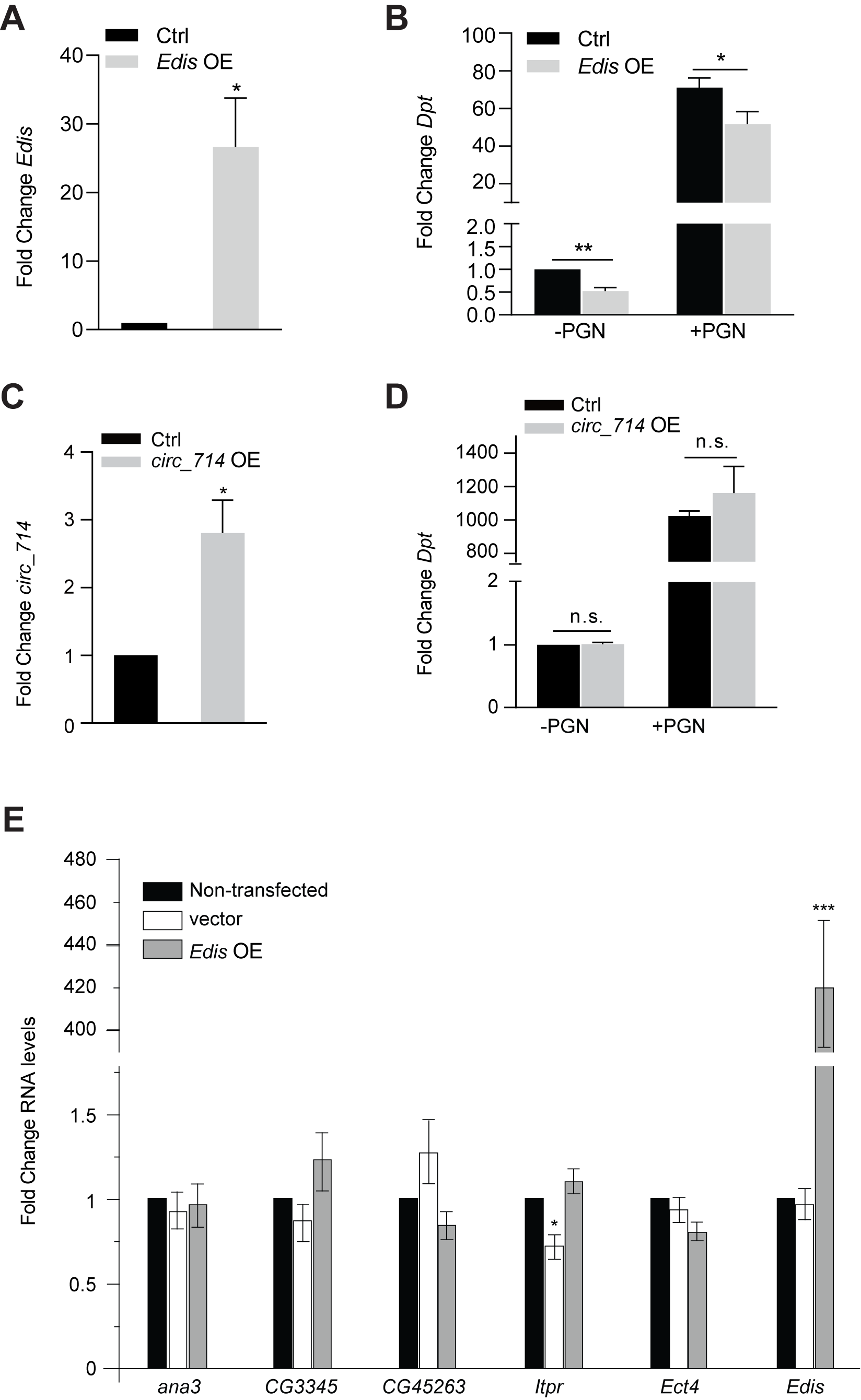

Supplement: S8 Fig — S2 cells were transfected with constructs expressing Edis (A-B) or circ_714 (C-D). Cells were treated with 20-HE and PGN. Levels of the Edis or circ_714 circular RNAs and the Dpt mRNA were measured and normalized to RpL32 (student t test, n = 3, * p<0.05; ** p<0.01). Cells transfected with an empty vector served as control. (E) Blast search against the Drosophila melanogaster genome using the Edis primer sequence pulled out a number of loci besides Ect4. To demonstrate specificity of the Edis primers, we chose the following criteria in selecting loci for further analysis: 1) 14 nt or more overlap; 2) the overlaps map to exons of annotated transcripts; and 3) the overlapping region is located within 3 nts of the 3’ end of the Edis primer. Edis overexpression does not affect expression of these genes (student t test, n = 3, * p<0.05; *** p<0.001). (TIF) [file pgen.1010429.s008.tif]

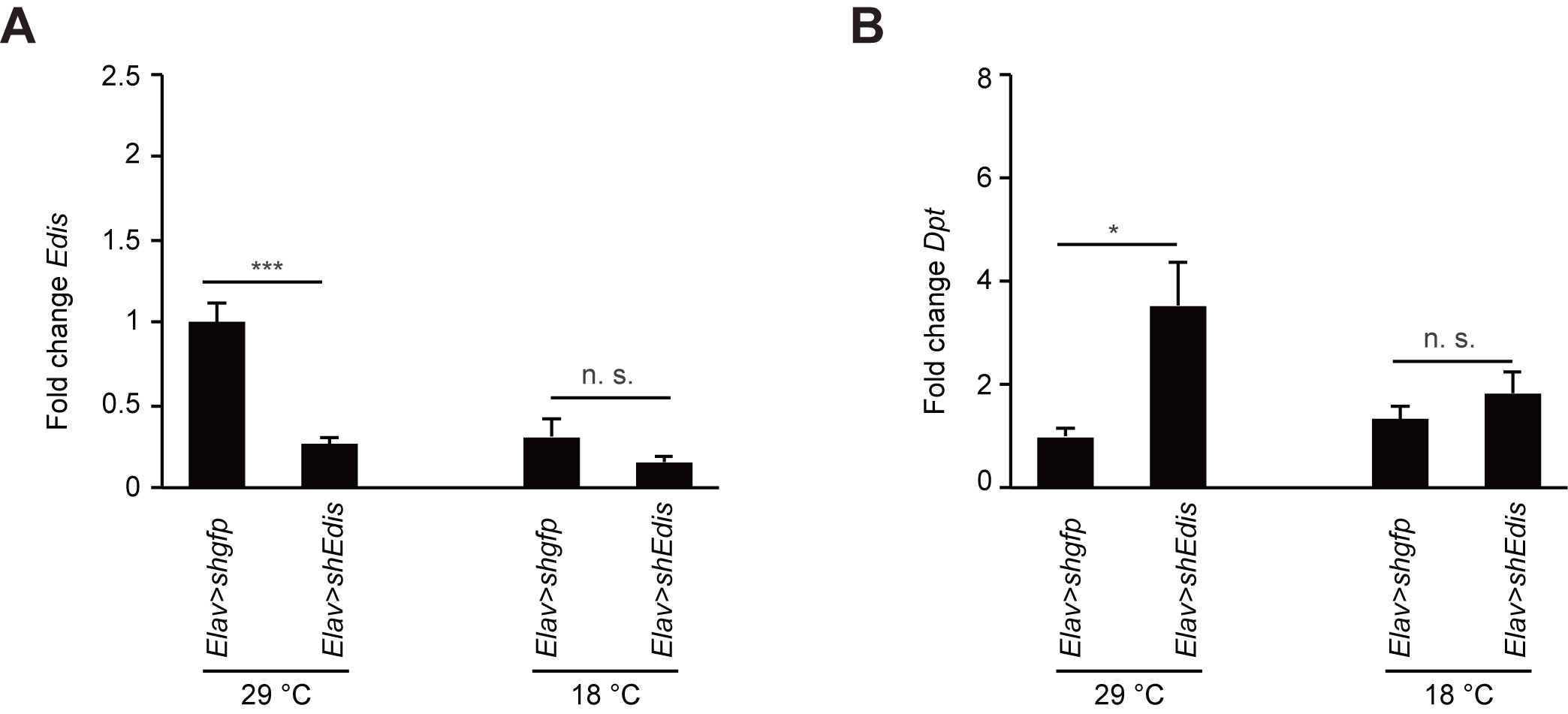

Supplement: S9 Fig — (A-B) Flies carrying the ubiquitously expressed daughterless-Gal4 driver and a temperature-sensitive Gal80 were crossed to UAS-shEdis or control shGFP flies. Fly crosses were kept at 18°C. Adult progeny were collected and either kept at 18°C or shifted to 29°C for 5 days to allow for shRNA transgene expression. Total RNAs were extracted and levels of the circular RNA Edis (A) and Dpt (B) were measured (student t test, n≥3, * p<0.05; *** p<0.001; ns, non-significant). (TIF) [file pgen.1010429.s009.tif]

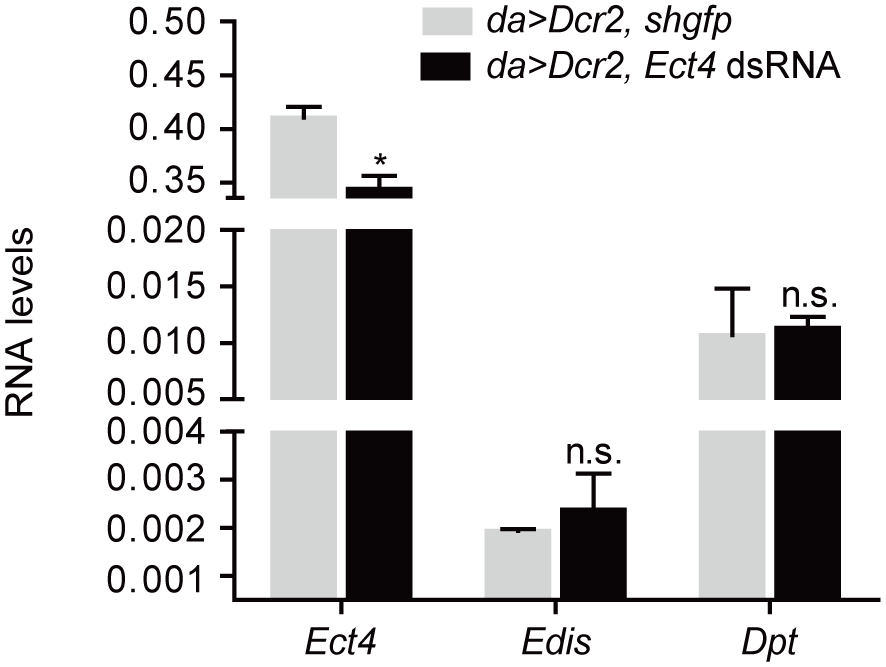

Supplement: S10 Fig — Flies carrying the ubiquitously expressed daughterless-Gal4 driver and a temperature-sensitive Gal80 were crossed to UAS-Dcr2; UAS-dsEct4 or control UAS-Dcr2; shGFP flies. Fly crosses were kept at 18°C. Adult progeny were shifted to 29°C for 5 days to allow for transgene expression. Total RNAs were extracted and levels of linear Ect4, circular Edis and Dpt transcripts were measured (student t test, n≥3, * p<0.05; ns, non-significant). (TIF) [file pgen.1010429.s010.tif]

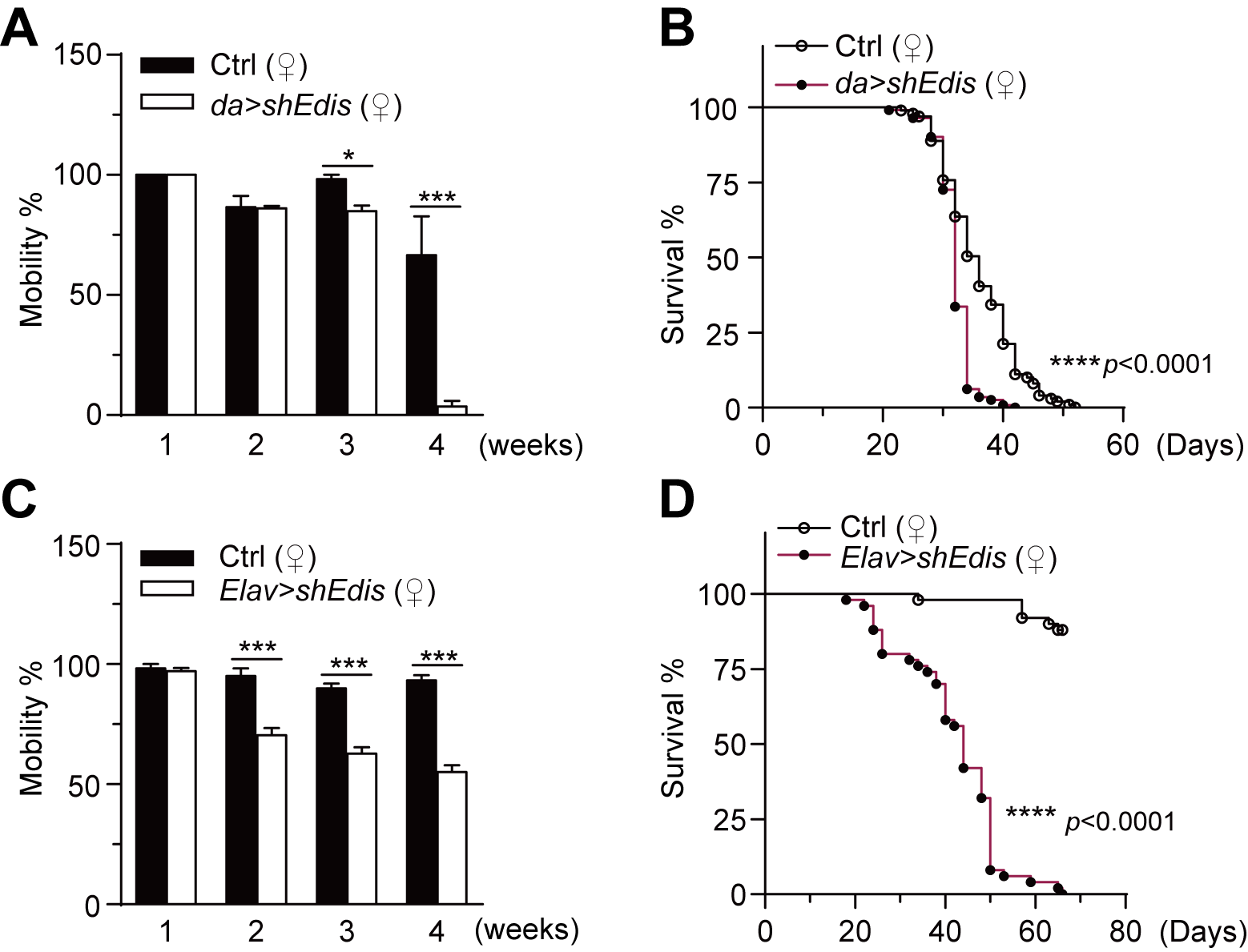

Supplement: S11 Fig — (A) female da>shEdis; Gal80ts flies and control da>shGFP; Gal80ts flies were kept at 29°C for indicated period of time (below). Flies in groups of 15 were placed into conical culture tubes and tapped to the bottom, and the percentage of flies that can climb over the 2-centimeter mark within 15 seconds was recorded and shown (student t test, n≥4, * p<0.05; *** p<0.001). (B) Female da>shEdis; Gal80ts flies and control da>shGFP; Gal80ts flies in multiple groups of 25 were kept at 29°C. Fly survival was recorded daily and plotted (log rank test, n≥6, **** p<0.0001). (C-D) The UAS-shEdis or control UAS-shGFP flies were crossed to the neuron-specific Elav-Gal4 driver line. Locomotor activity (C) and lifespan (D) of 1 to 4 week old female Elav>shEdis and control flies were measured and shown (student t test in C and log rank test in D, n = 4 to 8, *** p<0.001; **** p<0.0001). (TIF) [file pgen.1010429.s011.tif]

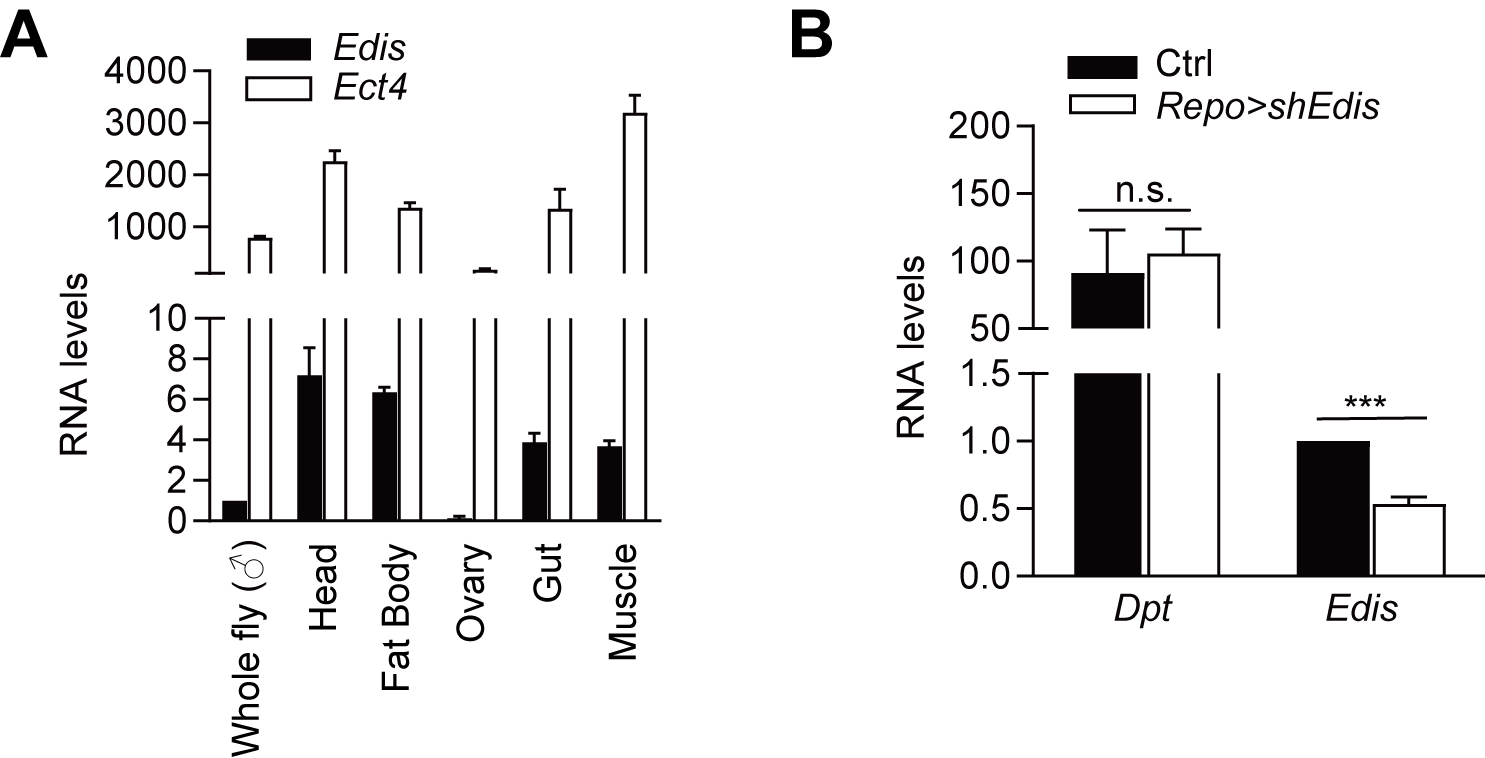

Supplement: S12 Fig — (A) Total RNA was extracted from either whole flies or various dissected tissues, and levels of Edis and Ect4 were measured by RT-qPCR and normalized to the control RpL32 mRNA (student t test, n = 3). (B) The UAS-shEdis or control UAS-shGFP flies were crossed to the glia-specific Repo-Gal4 driver line. Total RNA was prepared from dissected fly heads, and levels of the Dpt mRNA and Edis were measured (student t test, *** p<0.001; ns, non-significant). (TIF) [file pgen.1010429.s012.tif]

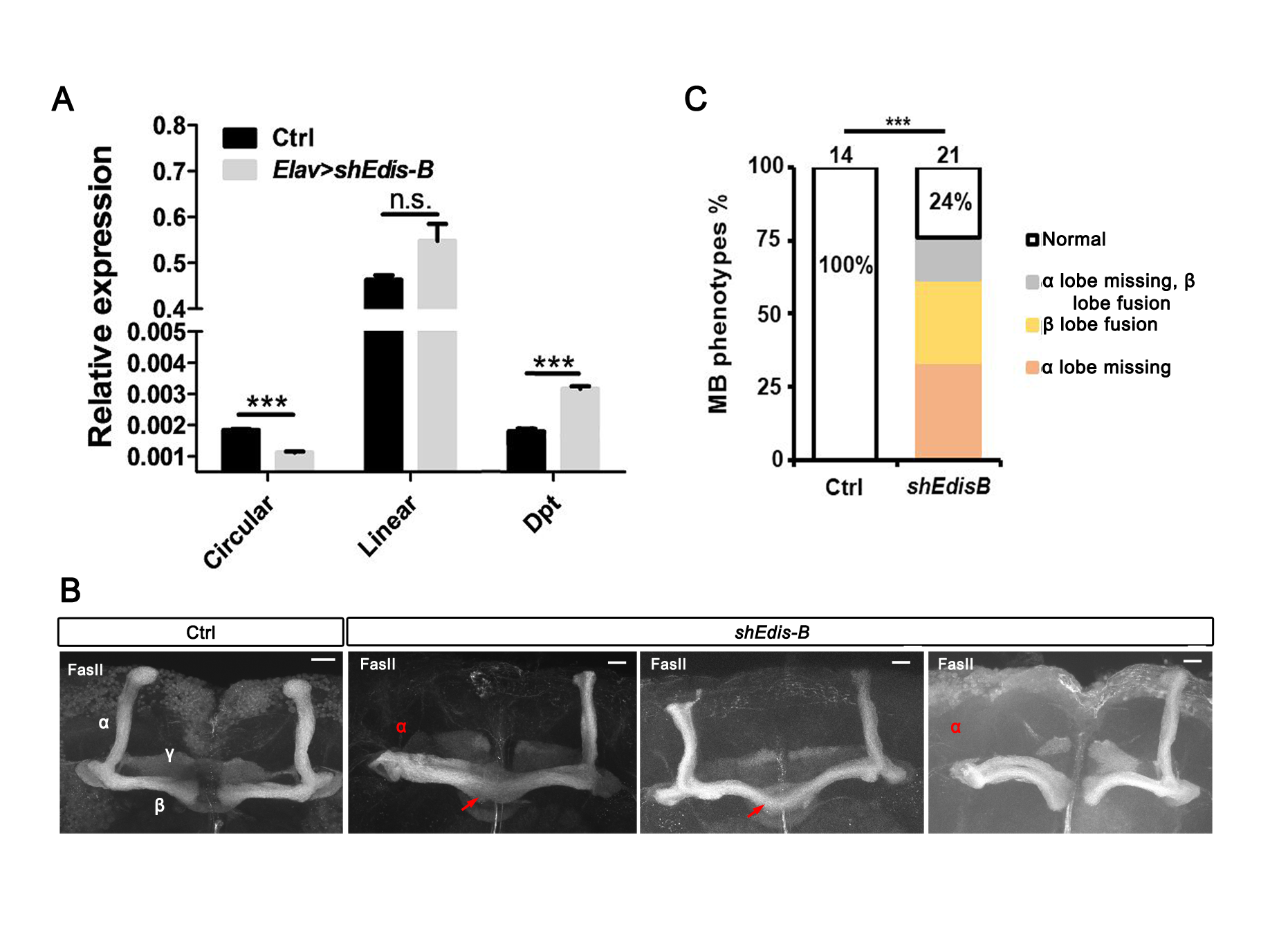

Supplement: S13 Fig — (A) Control UAS-shgfp or UAS-shEdis-B flies were crossed to the neuron-specific Elav-Gal4 driver animals. Total RNA samples were prepared from fly heads of the indicated genotypes. Levels of the Edis, Ect4 and Dpt transcripts were measured and normalized to rp49 (student t test, n≥3, *** p<0.001; ns, non-significant). (B) Shown are confocal images of dorsal anterior regions of adult brains with neuron-specific expression of control shgfp (left panel) or shEdis-B (middle and right panels) driven by the neuron-specific Elav-Gal4 driver. In control brain (left panel), anti-FasII antibody delineates the central complex as well as the vertical α and horizontal β and γ lobes of the mushroom bodies (MBs), with γ lobes showing weaker FasII signal, as indicated. Depletion of Edis resulted in a spectrum of severe morphological defects in the MBs, including missing (red circles) and/or fused lobes (red arrows). Scale bar: 20 μm. (C) Quantification of MB morphology phenotypes shown in B (Chi-squared test, sample numbers are shown on top, *** p<0.001). Percentage of animals displaying normal MB morphology is shown. (TIF) [file pgen.1010429.s013.tif]

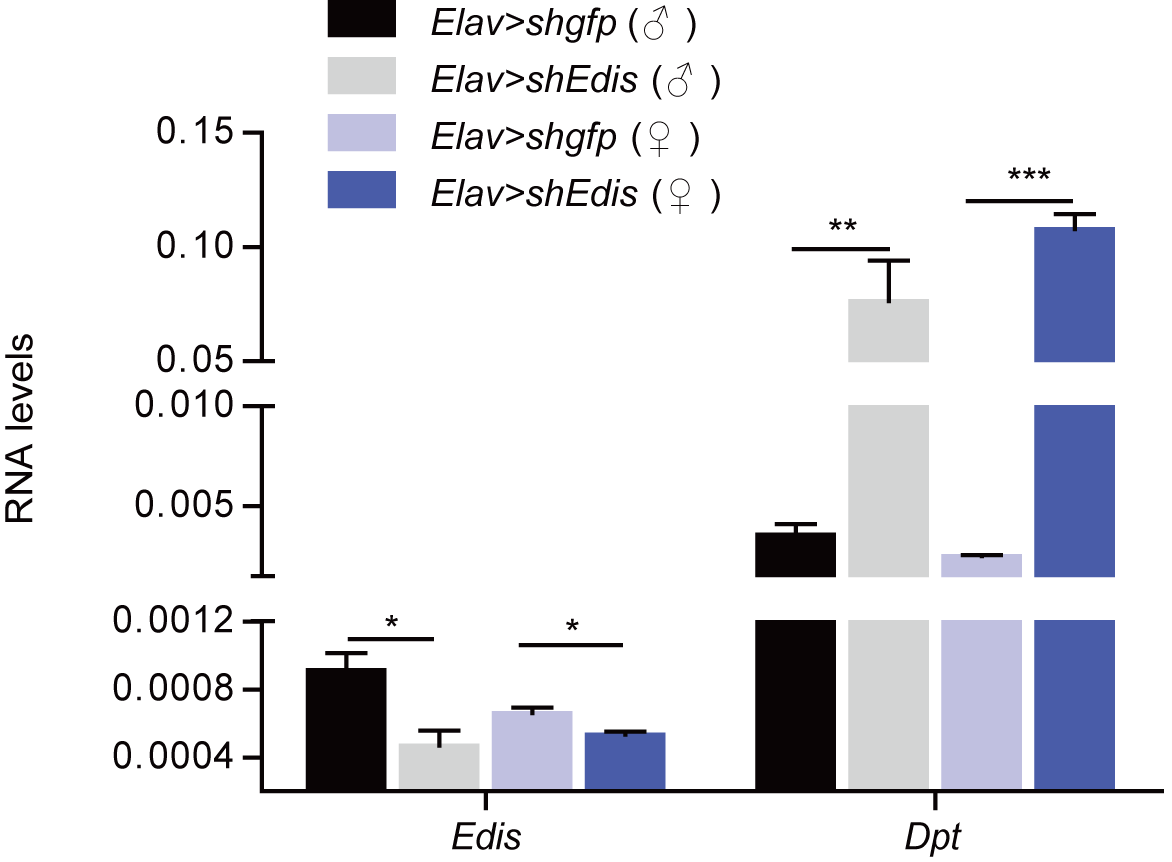

Supplement: S14 Fig — The UAS-shEdis or control UAS-shGFP flies were crossed to the neuron-specific Elav-Gal4 driver line. Levels of the circRNA Edis or the Dpt mRNA in male or female fly head samples were measured (student t test, n = 3, * p<0.05; ** p<0.01; *** p<0.001). (TIF) [file pgen.1010429.s014.tif]

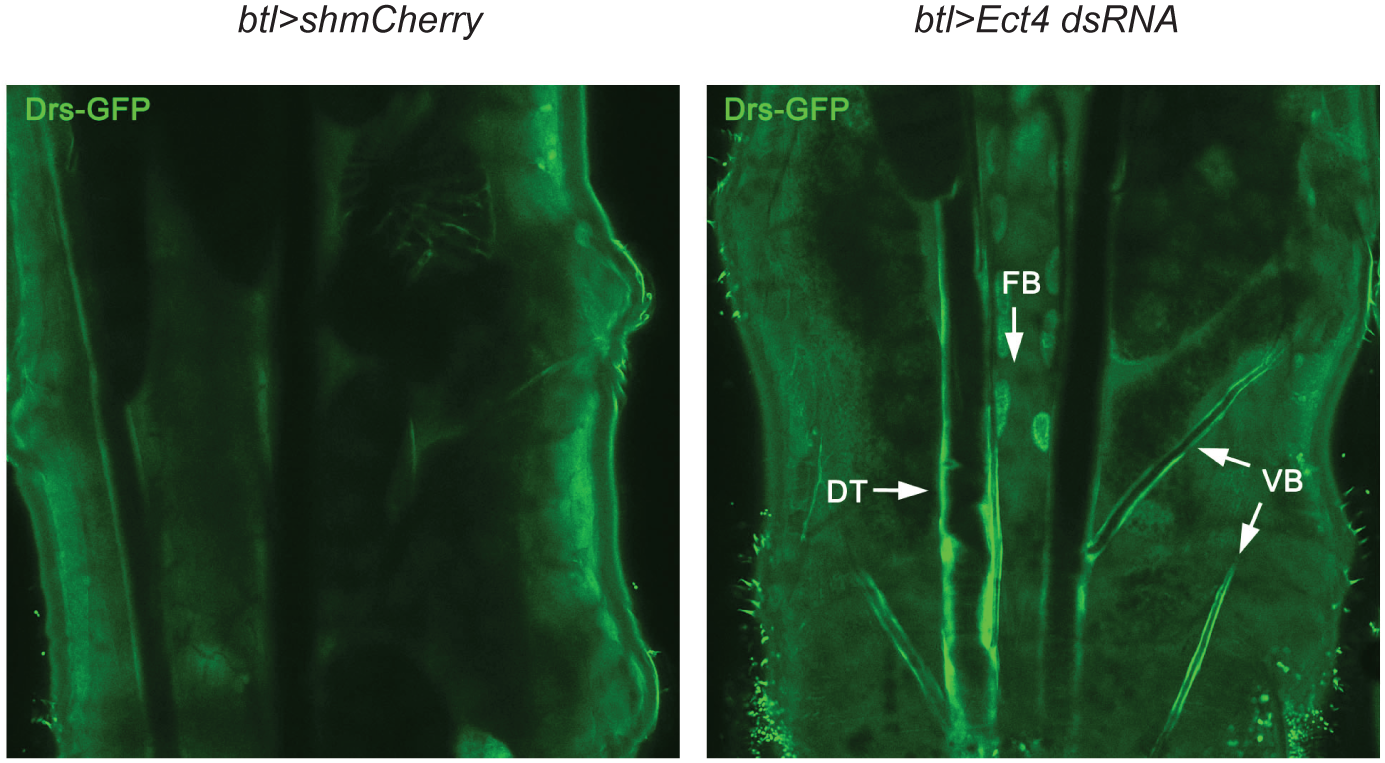

Supplement: S15 Fig — The control UAS-shmCherry or UAS-dsEct4 flies were crossed to the UAS-Dcr-2 breathless (btl)-Gal4 driver line. A Drosomycin (Drs) promoter driven GFP transgene was also present in the genetic background, to visualize the activation of antimicrobial peptide genes. Progeny larvae were fed with food containing Ecc15 for 24 hours. Levels of GFP signal in the trachea of third instar larvae were examined (n = 3). Representative images are shown. VB: visceral branch, DT: dorsal trunk, FB: fat body. (TIF) [file pgen.1010429.s015.tif]

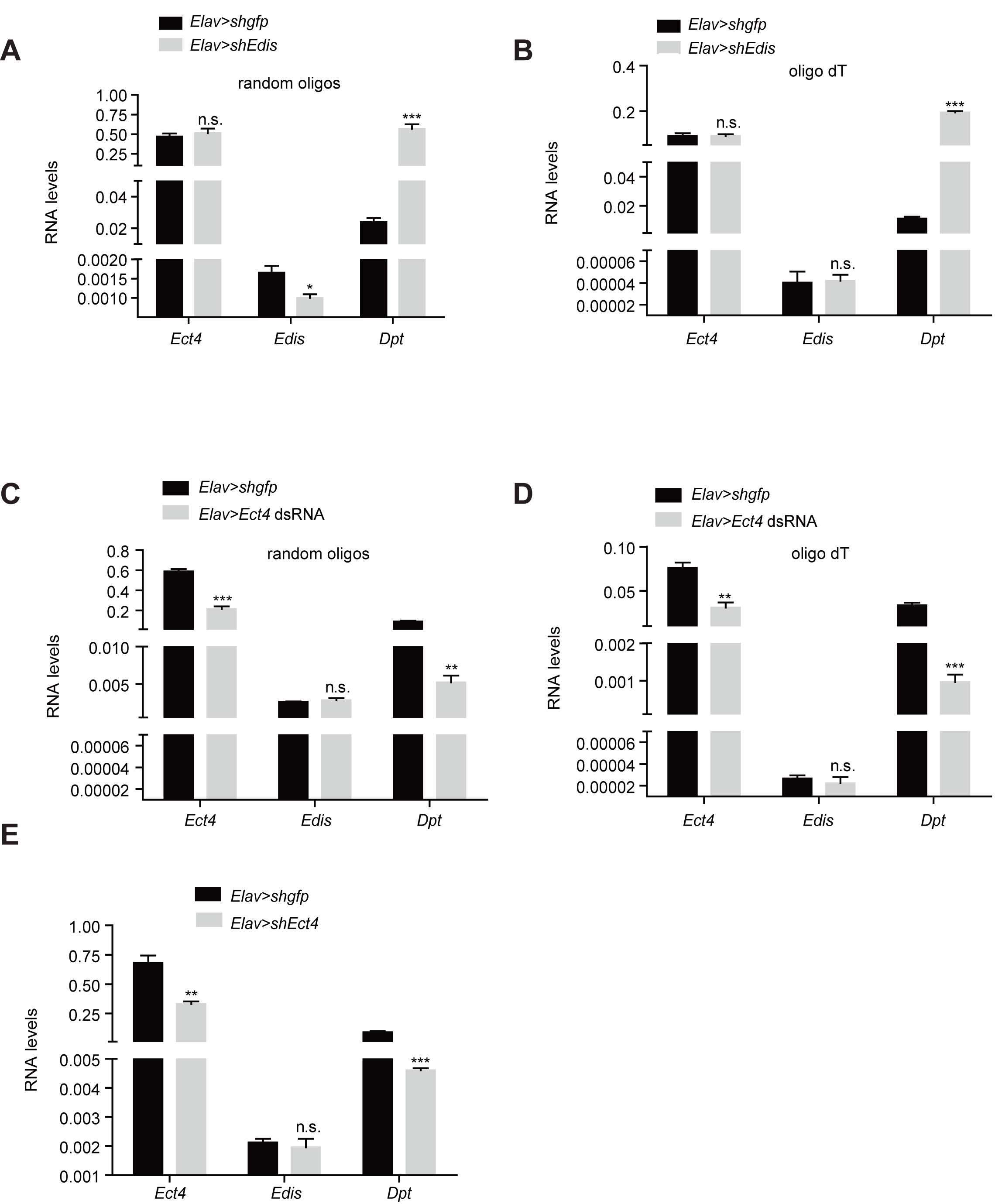

Supplement: S16 Fig — The UAS-shEdis (A-B) or UAS-dsEct4 (C-D) flies were crossed to the UAS-Dcr-2 Elav-Gal4 driver line. Total RNAs were extracted from male fly head samples and reverse transcribed using either random primers (A,C) or oligo dT (B,D). Levels of the circRNA Edis, linear Ect4 or the Dpt mRNA were measured (student t test, n = 3, * p<0.05; ** p<0.01; *** p<0.001; ns, non-significant). Samples from a cross between UAS-shGFP and UAS-Dcr-2 Elav-Gal4 driver line serve as controls. As expected, we detected consistent changes in levels of the linear Ect4 and Dpt mRNAs regardless of the type of oligos employed in the reverse transcription systems, whereas changes in levels of the circRNA Edis that were previously detected in random primer-based reverse transcription reactions were no longer obvious in oligo d(T)-based reverse transcription reactions, as the circRNA Edis was not expected to be efficiently reverse transcribed using oligo d(T). (E) The UAS-shGFP or UAS-shEct4 flies were crossed to the Elav-Gal4 driver line. Total RNAs were extracted from male fly head samples and reverse transcribed using either random primers. Levels of the circRNA Edis, linear Ect4 or the Dpt mRNA were measured (student t test, n = 3, *** p<0.001; ns, non-significant). (TIF) [file pgen.1010429.s016.tif]

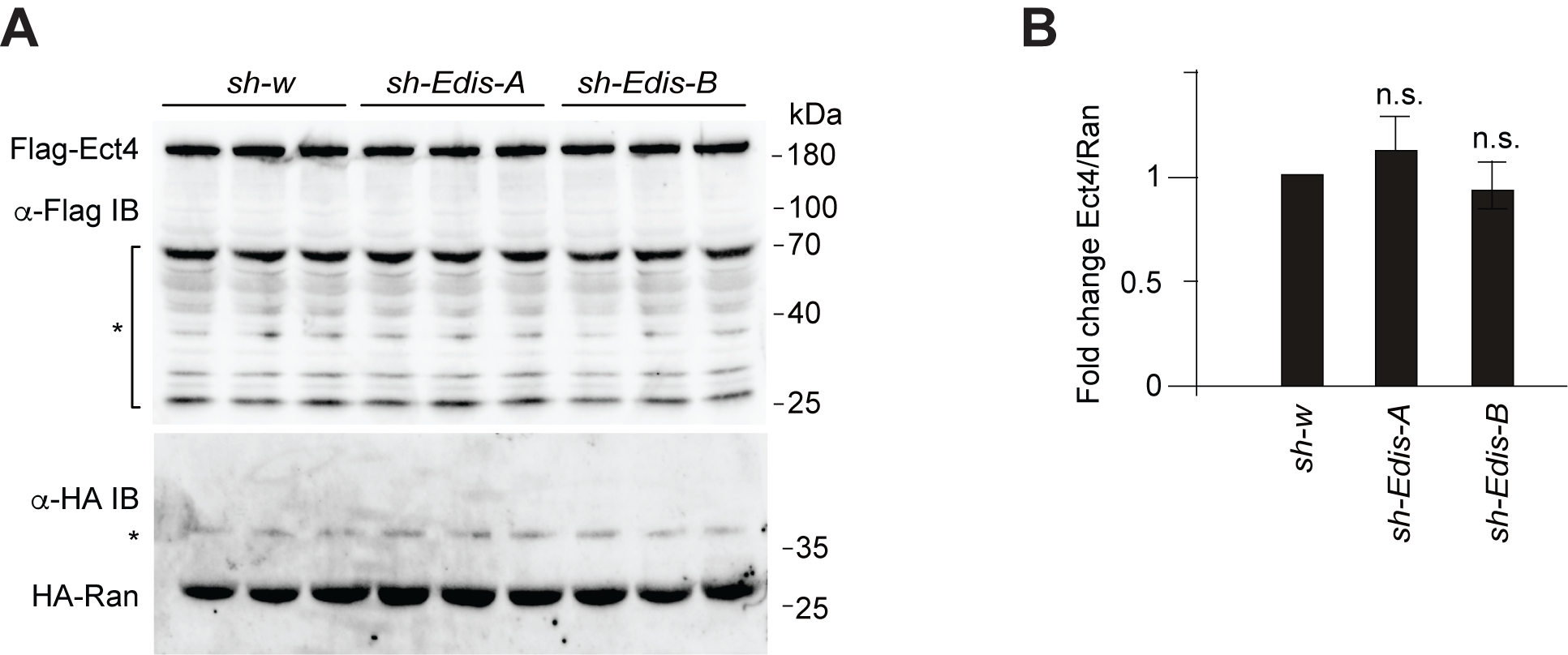

Supplement: S17 Fig — (A) S2 cells stably transfected with the shEdis or control shRNA constructs were transfected with Flag-Ect4 and HA-Ran expression constructs. Cells were treated with copper to induce transgene expression, and subjected to immunoblot assay using anti-Flag (upper panel) and anti-HA (lower panel) antibodies. (B) The ratio of Flag-Ect4/HA-Ran in A was quantified and shown (student t test, n = 3, ns, non-significant). (TIF) [file pgen.1010429.s017.tif]

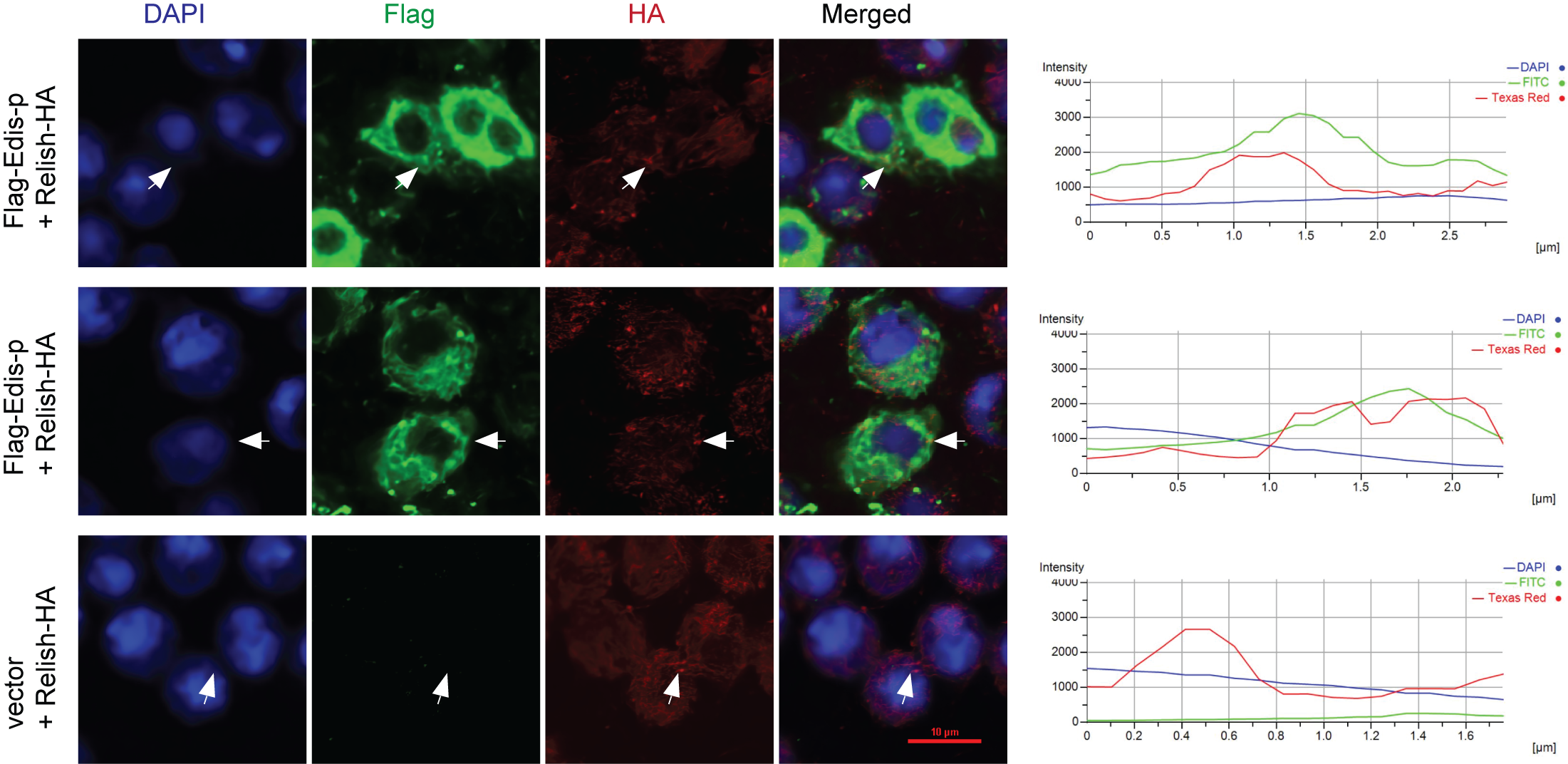

Supplement: S18 Fig — S2 cells were transfected with various combinations of pMT, pMT-Flag-Edis-p and pMT-Relish-HA constructs, as indicated on the left. Epitope-tagged proteins were visualized by staining with antibodies against Flag (green) and HA (red) epitopes. Nuclei were visualized by DAPI staining (blue). Columns from left to right show DAPI, Flag, HA signals and overlay of the three channels, respectively. Arrows point to co-localization between Flag-Edis-p and Relish-HA. Shown on the right are quantifications of signals of various channels along a line that crosses the region pointed by the arrows. (TIF) [file pgen.1010429.s018.tif]

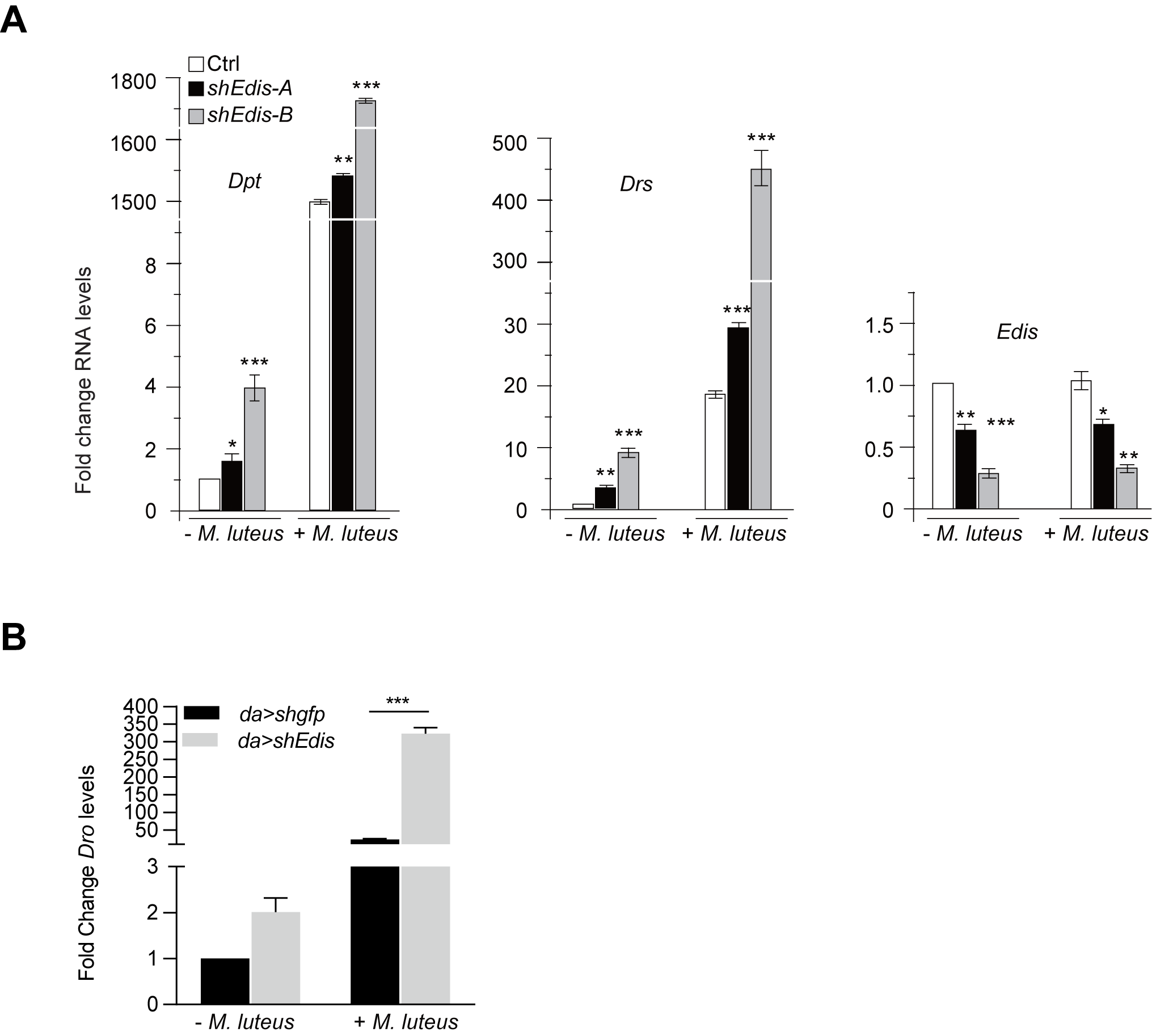

Supplement: S19 Fig — (A) S2 cells were transfected with two independent shRNA constructs (sh-Edis-A or -B) targeting the back-spliced exon junction of Edis or a control (Ctrl) shRNA against the white gene. Cells were treated with 20-hydroxyecdysone (20-HE) and subsequently with (+) or without (-) the Gram-positive bacterial M. luteus. Levels of Diptericin (Dpt), Drosomycin (Drs) mRNAs and Edis were measured by RT-qPCR and normalized to RpL32 (student t test, n = 3, * p<0.05; ** p<0.01; *** p<0.001). (B) Flies carrying the ubiquitously expressed daughterless-Gal4 driver and a temperature-sensitive Gal80 were crossed to UAS-shEdis or control shGFP flies. Fly crosses were kept at 18°C. Adult progeny were collected and shifted to 29°C for 5 days to allow for shRNA transgene expression. Flies were either left un-infected or pricked with a needle previously dipped into a concentrated culture of the Gram-positive bacteria M. luteus. Flies were collected 24 hours later and levels of the circular RNA Edis and Drosomycin (Drs) were measured (student t test, n = 3; n = 1 for non-infected da>shgfp sample, *** p<0.001). (TIF) [file pgen.1010429.s019.tif]
